# From root to fruit: RNA-Seq analysis shows that arbuscular mycorrhizal symbiosis may affect tomato fruit metabolism

**DOI:** 10.1186/1471-2164-15-221

**Published:** 2014-03-21

**Authors:** Inès Zouari, Alessandra Salvioli, Matteo Chialva, Mara Novero, Laura Miozzi, Gian Carlo Tenore, Paolo Bagnaresi, Paola Bonfante

**Affiliations:** Department of Life Sciences and Systems Biology, University of Turin, Viale Mattioli 25, 10125 Turin, Italy; Institute of Plant Virology-IVV-CNR, Strada Delle Cacce, 73, 10135 Turin, Italy; Department of Pharmacy, University of Naples Federico II, via D. Montesano 49, 80131 Naples, Italy; Consiglio per la Ricerca e la Sperimentazione in Agricoltura, Genomics Research Centre, via S. Protaso, 302 I, 29017 Fiorenzuola d’Arda, PC, Italy

**Keywords:** Arbuscular mycorrhizal fungi, Tomato, RNA sequencing, Fruit gene expression, Systemic effects

## Abstract

**Background:**

Tomato (*Solanum lycopersicum*) establishes a beneficial symbiosis with arbuscular mycorrhizal (AM) fungi. The formation of the mycorrhizal association in the roots leads to plant-wide modulation of gene expression. To understand the systemic effect of the fungal symbiosis on the tomato fruit, we used RNA-Seq to perform global transcriptome profiling on Moneymaker tomato fruits at the turning ripening stage.

**Results:**

Fruits were collected at 55 days after flowering, from plants colonized with *Funneliformis mosseae* and from control plants, which were fertilized to avoid responses related to nutrient deficiency. Transcriptome analysis identified 712 genes that are differentially expressed in fruits from mycorrhizal and control plants. Gene Ontology (GO) enrichment analysis of these genes showed 81 overrepresented functional GO classes. Up-regulated GO classes include photosynthesis, stress response, transport, amino acid synthesis and carbohydrate metabolism functions, suggesting a general impact of fungal symbiosis on primary metabolisms and, particularly, on mineral nutrition. Down-regulated GO classes include cell wall, metabolism and ethylene response pathways. Quantitative RT-PCR validated the RNA-Seq results for 12 genes out of 14 when tested at three fruit ripening stages, mature green, breaker and turning. Quantification of fruit nutraceutical and mineral contents produced values consistent with the expression changes observed by RNA-Seq analysis.

**Conclusions:**

This RNA-Seq profiling produced a novel data set that explores the intersection of mycorrhization and fruit development. We found that the fruits of mycorrhizal plants show two transcriptomic “signatures”: genes characteristic of a climacteric fleshy fruit, and genes characteristic of mycorrhizal status, like phosphate and sulphate transporters. Moreover, mycorrhizal plants under low nutrient conditions produce fruits with a nutrient content similar to those from non-mycorrhizal plants under high nutrient conditions, indicating that AM fungi can help replace exogenous fertilizer for fruit crops.

**Electronic supplementary material:**

The online version of this article (doi:10.1186/1471-2164-15-221) contains supplementary material, which is available to authorized users.

## Background

Tomato (*Solanum lycopersicum*), like other solanaceous species, establishes a beneficial root symbiosis with arbuscular mycorrhizal (AM) fungi of the Glomeromycota [1]. The plant roots accommodate the AM fungi, which improve nutrient uptake of their plant hosts, and, in turn, receive fixed carbon from the plant [2], with a reciprocal reward for the best partner [3]. Furthermore, research on tomato mycorrhizal roots has identified plant genes as functional markers for AM symbiosis and shown that they are expressed in different cell types [4, 5]. In addition to the expected effects on roots, mycorrhization also affects gene expression in tomato shoots and leaves, modulating genes involved in diverse metabolic processes such as defence, transport and hormonal metabolism [6, 7]. A pilot microarray experiment also demonstrated a systemic effect on the fruit transcriptome [8].

Tomato represents an excellent model plant for understanding the long-distance effects of AM fungi on organs other than roots, as it exhibits substantial increases in growth and fruit yield in response to mycorrhization [9–11]. In addition, extracts of tomato fruit produced from mycorrhizal plants showed an anti-oestrogenic activity likely attributable to their higher contents of lycopene [12].

Analysis of the tomato (*Solanum lycopersicum*) genome [13] has showed that many tomato genes are specifically related to the evolution of fleshy fruits, and that intensive breeding has selected some agriculturally relevant traits. Recent work has characterized the molecular mechanisms that govern the transition of tomato fruit from a green to a fully red ripe fruit, *via* the intermediate stages termed breaker, turning and pink. Tomato ripening involves increased levels of aromatic compounds and carotenoid pigments, softening of the cell wall and decreased photosynthetic activity. Ethylene, the key hormone regulating ripening in climacteric fruit, controls most of these changes through a complex mechanism [14, 15]. Accumulating data have helped to define the roles played by specific genes [16] in specific fruit cell types [17], and also have extended the analysis to tomato epigenetic and proteomic dynamics [18].

To better explore the intersection between mycorrhization and fruit development in tomato, we used RNA-Seq to profile transcription changes [19] in fruits originating from mycorrhizal plants, a condition which has so far not been analysed in a crop plant of high agricultural interest. We found that the fruits of mycorrhizal plants show regulation of transcripts characteristic of climacteric fleshy fruits and a core set of genes typically modulated by symbiosis. Moreover, our quantitative analyses showed that fruits from mycorrhizal and non-mycorrhizal fertilized plants have comparable levels of carotenoids, phenols, and minerals (i.e. phosphorus, sulfur and potassium) indicating that the AM symbiosis benefits fruit quality. Thus, AM fungi can serve as surrogates for fertilization, providing similar benefits at the root and fruit levels.

## Results

### Plant sampling for RNA-Seq

**Figure 1:**
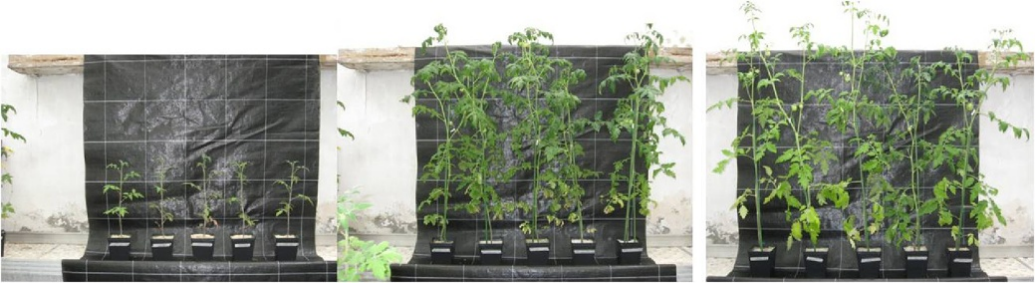
**Development of tomato plants at 77 days after transplanting.** Left: non-inoculated, low-fertilized plants; middle: non-inoculated highly-fertilized control plants (CONT); right: Mycorrhizal low-fertilized plants (MYC).

To examine the transcriptome of tomato fruits from mycorrhizal plants, we collected fruits from plants colonized by the AM fungus *Funneliformis mosseae* (previously named *Glomus mosseae*) and from non-inoculated plants. AM fungi enhance nutrient uptake by their hosts [20]; to control for nutrition-dependent effects, we provided increased nutrients to the non-inoculated plants, to produce two groups of plants that have a similar phenology and nutritional status. Thus, we watered the mycorrhizal plants (MYC plants) with a low-phosphorus (P), nitrogen (N) and sulfur (S) solution, which allowed high mycorrhization levels and healthy plant growth [8, 21] but we watered the control, non-mycorrhizal plants (CONT plants) with higher concentrations of P, S and N. The MYC and CONT tomato plants grown under these different conditions showed similar growth (Figure 1) and similar P, S and K contents in their fruits (Additional file 1: Table S1), suggesting that the mycorrhizal fungus successfully compensated for the low fertilization treatment. We also measured some phenological features of the plant, including fruit yield, and found that the MYC and CONT fruits showed comparable values (Additional file 1: Table S2). The one exception was fruiting time, which was longer for mycorrhizal plants, leading to the production of more fruit. By contrast, plants that were not inoculated with *F. mosseae* but were fertilized with a similar, low-P and -N regimen to MYC plants were strongly affected by the nutrient limitations, showing impaired growth (Figure 1) and delayed fruit production, leading to the production of fewer fruit.

To assess the effect of mycorrhization on a key stage of ripening, we collected fruits at the turning stage, when the green color starts to change on 10 to 30 percent of the fruit surface, indicating the beginning of carotenoid biosynthesis [22]. Because the MYC and CONT plants have comparable vegetative periods, we harvested fruits from both conditions at 55 days after flowering. The MYC plants showed a colonization frequency (F) of 65% and a mycorrhizal intensity (M) of 36% in the root system (Additional file 2: Figure S1). We observed no traces of fungal colonization in the CONT roots. Two biological replicates for each condition were collected, following the current RNA-Seq standards (http://encodeproject.org/ENCODE/protocols/dataStandards/ENCODE_RNAseq_Standards_V1.0.pdf). The samples were then used for deep single-end sequencing (read length: 100 bp).

### Analyses of RNA-Seq data: read number, transcriptome coverage and total expressed genes

To identify differentially expressed transcripts, we first filtered the sequences, estimated the transcriptome coverage, and identified expressed genes. The raw reads (100 bases, single-end) obtained from Illumina HIseq (FASTERIS Co. Geneva, Switzerland) were filtered (Illumina passed-filter call) and further checked for sequence contaminants with the fastQC application. Contaminant-free, filtered reads ranging from 11 to 16 million for each sample (Table 1) were mapped with Bowtie/TopHat to the tomato genome sequence SL2.40 (ITAG2.3) [13]. Raw read counts were obtained from alignment files by counting with HTSeq software. Based on the sum of transcript lengths, as reported in the current SL2.40 annotation (41,393,518 bp; which does not currently account for transcript isoforms), we reached an average transcriptome coverage of 23x for each replicate. An RPKM (Reads per Kilobase per Million reads) cutoff value of 0.1 was set to declare a locus expressed, resulting in 21,530 loci above the expression threshold. We also performed GO functional analysis to identify the biological processes that were most represented by the expressed genes. The GOSLIM terms for biological process assignment to the total expressed genes showed that cellular protein modification, carbohydrate metabolic, transmembrane transport, translation and lipid metabolic processes were the most-represented biological categories (Additional file 2: Figure S2).

**Table 1:** **Read number and alignment summary**

Sample	Total number of filtered reads	Total alignments	Total unique aligned reads	Total aligned bases	Uniquely aligned mapping reads
CONT_1	14,660,414	13,136,222	12,790,191	1,038,315,933	12,552,493
CONT_2	16,303,483	14,602,025	14,229,313	1,141,477,858	13,973,208
MYC_1	12,053,452	10,748,794	10,510,262	860,932,723	10,336,271
MYC_2	11,715,434	10,222,971	9,939,712	814,018,186	9,740,416

### Differentially expressed genes and overrepresented categories

To identify differentially expressed genes (DEGs), we used the R package DESeq to evaluate the significance of differences in expression and to control for false discovery rate (FDR). Pearson correlation coefficients among biological replicates were subjected to the same treatment and were all above 0.95. The FDR threshold was set to 0.1 and gene dispersion values were calculated by fitting a curve according to the DESeq “fit-only” mode. Using these criteria, we identified 712 DEGs (Additional file 3: Table S3), including 458 up-regulated genes and 254 down-regulated genes. We also used MA plot analysis to examine the magnitude distribution of the significantly regulated genes comparing the expression level to the log-transformed fold-change between MYC and CONT (Additional file 2: Figure S3). Among the DEGs, the 5 most highly-expressed genes (regardless of the growth condition) were a fruit-specific protein, Linoleate 9S-lipoxygenase B, the acid beta-fructofuranosidase and two heat shock proteins 90 and 70 (Table 2). These genes affect important fruit processes like ripening, stress responses, and lipid and carbohydrate biosynthesis.

**Table 2:** **List of the five most strongly expressed genes**

Gene ID	ITAG2.3_descriptions	Description SL2.40	Mean read number	Log _2_ ratio MYC vs CONTR
Solyc07g049140.2.1	Metallocarboxypeptidase inhibitor	Fruit-specific protein	200,347	-1,097
Solyc01g099190.2.1	Lipoxygenase	Linoleate 9S-lipoxygenase B	149,420	-1,178
Solyc03g083910.2.1	Acid beta-fructofuranosidase	Acid beta-fructofuranosidase	59,651	-1,635
Solyc07g064170.2.1	Pectinesterase	Pectinesterase 1	55,636	-1,154
Solyc06g036290.2.1	Heat shock protein 90		29,385	1,066
Solyc04g011440.2.1	Heat shock protein 70 isoform 3	heat shock protein	26,164	0,826

The listed genes represent the five most expressed genes among the DEGs. The selection was based on the mean number of reads in MYC and CONT fruits.

### GO annotation of differentially expressed genes

**Figure 2:**
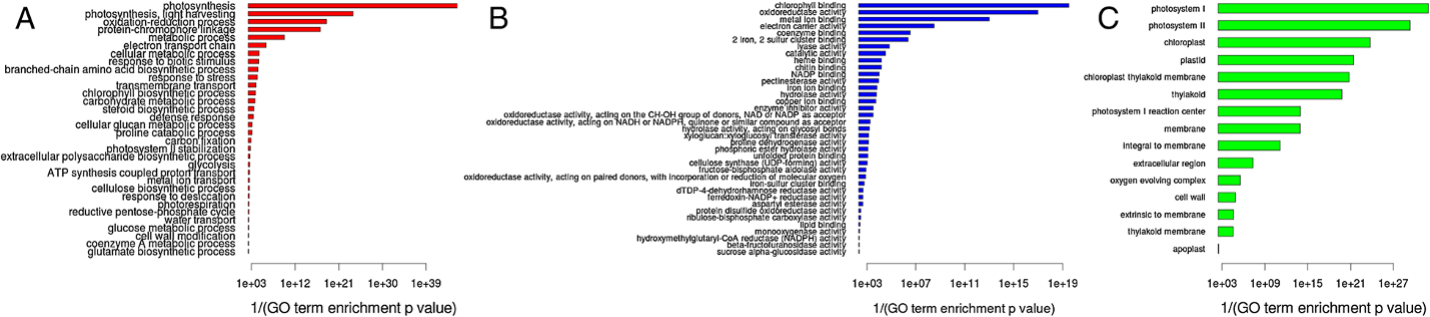
**GO enrichment in DEGs.** The 81 enriched GO terms (y axis labels; FDR threshold ≤ 0.1) associated to DEG, as assessed compared with all expressed genes (RPKM > 0.1), are plotted along the reciprocal of Enrichment p value (x axis) as calculated with the Goseq R package. Panel **A**: Biological Process; panel **B**: Molecular Function; panel **C**: Cell Compartment.

We used GO enrichment analysis to identify the major gene groups affected by mycorrhization. As in all RNA-Seq experiments, the variable length of transcripts may produce biases in the data [23]; therefore, we analyzed GO enrichment *via* the Goseq R package, which was specifically designed to limit bias in the data [24]. We found 81 GO terms that were over-represented (FDR ≤ 0.1) in response to mycorrhization (Figure 2). The biological processes corresponding to the lowest p-values, in decreasing order of importance, were “photosynthesis” (p = 2.66E-43), “photosynthesis light harvesting” (p = 4.48E-24), “protein-chromophore linkage” (p = 2.45E-17), “oxidation-reduction process” (p = 1.74E-13). Other relevant over-represented categories included: “response to biotic stimulus”, “transmembrane transport” and “carbohydrate metabolic process” (Figure 2).

### Gene regulation in tomato fruit of mycorrhizal *versus*fertilized plants

Examination of GO terms suggested that a large part of the modulated tomato transcriptome is devoted to control of the main metabolic processes (i.e. photosynthesis and photorespiration) and to ripening (from cell-wall to secondary metabolites). For example, we identified 78 genes involved in oxidation-reduction processes, 54 in metabolic processes, 49 in photosynthesis, 43 with a catalytic activity, 30 involved in transport process, 24 transcription factors, 20 with a role in carbohydrate metabolism, 19 stress-responsive genes, 11 hormone-related genes and 51 unknown proteins corresponding to 10.95%, 7.58%, 6.88%, 6%, 4.17%, 3.37%, 2.8%, 2.66%, 1.52%, and 7.12% of the regulated gene core set, respectively. In the following sections, we focus on a subset of these genes.

### Photosynthesis- and photorespiration-related processes

We identified a substantial number of genes involved in different steps of photosynthesis as up-regulated in MYC fruit (Table 3). Some of these up-regulated genes encode proteins of photosystem II and photosystem I, and others are involved in light-dependent reactions (e.g. cytochrome b6f, plastocyanins, ATP synthases and NADP reductases). Widespread up-regulation was also observed for genes related to the light-independent reactions or the Calvin cycle: two Ribulose bisphosphate carboxylases, one phosphofructokinase family protein, four fructose-1-6-bisphosphatases class 1 and three fructose-bisphosphate aldolases.

**Table 3:** **Mycorrhiza-regulated genes involved in photosynthesis and related metabolisms in turning tomato fruit**

ITAG2.3_descriptions	Description SL2.40	Gene ID	Log _2_ ratio MYC vs CONTR
**Photosystem I**			
Chlorophyll a/b binding protein	Chlorophyll a-b binding protein 1D	Solyc02g071000.1.1	Inf
Chlorophyll a-b binding protein 3C-like	Chlorophyll a-b binding protein 3C	Solyc03g005780.1.1	5,75
Chlorophyll a-b binding protein 37, chloroplastic	Chlorophyll a-b binding protein 5	Solyc12g006140.1.1	4,11
Chlorophyll a/b binding protein	Chlorophyll a-b binding protein 1B	Solyc02g070950.1.1	3,35
Chlorophyll a/b binding protein	Chlorophyll a-b binding protein 1B	Solyc02g071010.1.1	3,22
Photosystem I reaction center subunit VI-1, chloroplastic		Solyc06g066640.2.1	2,92
Chlorophyll a-b binding protein 13, chloroplastic	Chlorophyll a-b binding protein 13	Solyc12g011450.1.1	2,76
Chlorophyll a/b binding protein	Chlorophyll a-b binding protein 1B	Solyc02g071030.1.1	2,68
Photosystem I reaction center subunit		Solyc08g013670.2.1	2,63
Chlorophyll a/b binding protein	Chlorophyll a-b binding protein 1B	Solyc02g070940.1.1	2,61
Chlorophyll a-b binding protein 6A, chloroplastic	Chlorophyll a-b binding protein 6A	Solyc05g056070.2.1	2,61
Chlorophyll a-b binding protein 4, chloroplastic	Chlorophyll a-b binding protein 4	Solyc07g047850.2.1	2,54
Photosystem I reaction center subunit V		Solyc07g066150.1.1	2,35
Chlorophyll a-b binding protein 8, chloroplastic	Chlorophyll a-b binding protein 8	Solyc10g007690.2.1	2,23
Photosystem I reaction center subunit XI		Solyc06g082950.2.1	1,61
Photosystem I reaction center subunit III		Solyc02g069460.2.1	1,31
Photosystem I reaction center subunit VI-1, chloroplastic		Solyc03g120640.2.1	1,23
Photosystem I reaction center subunit IV A		Solyc06g083680.2.1	1,10
Photosystem I reaction center subunit XI		Solyc06g082940.2.1	2,48
Photosystem I reaction center subunit III		Solyc02g069450.2.1	2,41
Photosystem I reaction center subunit II	Photosystem I reaction center subunit II	Solyc06g054260.1.1	2,02
Photosystem I reaction center subunit IV A		Solyc09g063130.2.1	1,73
**Photosystem II**			
Chloroplast photosystem II-associated protein	Photosystem II 22 kDa protein	Solyc06g060340.2.1	5,06
Photosystem II reaction center psb28 protein		Solyc09g064500.2.1	2,53
Photosystem II reaction center W protein		Solyc06g084050.2.1	2,37
Chlorophyll a-b binding protein, chloroplastic	Chlorophyll a-b binding protein CP24 10A	Solyc01g105030.2.1	2,19
Chlorophyll a-b binding protein, chloroplastic	Chlorophyll a-b binding protein CP24 10B	Solyc01g105050.2.1	2,07
Chloroplast photosystem II subunit X (Fragment)		Solyc05g025600.1.1	1,97
Oxygen-evolving enhancer protein 2, chloroplastic	Oxygen-evolving enhancer protein 2	Solyc07g044860.2.1	1,58
Oxygen-evolving enhancer protein 3	Photosystem II oxygen-evolving complex protein 3	Solyc02g079950.2.1	1,16
Photosystem II reaction center W protein		Solyc09g065910.1.1	0,84
Oxygen-evolving enhancer protein 1 of photosystem II		Solyc02g090030.2.1	1,51
Oxygen-evolving enhancer protein 1 of photosystem II	Oxygen-evolving enhancer protein 1	Solyc02g065400.2.1	1,24
**Chlorophyll biosynthetic process**			
Geranylgeranyl reductase	Geranylgeranyl reductase	Solyc03g115980.1.1	2,20
Magnesium-protoporphyrin IX monomethyl ester		Solyc10g077040.1.1	2,10
Magnesium chelatase H subunit		Solyc04g015750.2.1	1,11
Magnesium-protoporphyrin ix methyltransferase		Solyc03g118240.2.1	1,09
Light dependent NADH:protochlorophyllide oxidoreductase 1	Light dependent NADH:protochlorophyllide oxidoreductase 1	Solyc12g013710.1.1	2,43
Chlorophyllide a oxygenase		Solyc06g060310.2.1	1,92
**Calvin cycle**			
Ribulose bisphosphate carboxylase small chain	Ribulose bisphosphate carboxylase small chain 1	Solyc02g063150.2.1	2,52
Ribulose bisphosphate carboxylase small chain	Ribulose bisphosphate carboxylase small chain 2A	Solyc03g034220.2.1	2,25
**Calvin, others**			
Phosphofructokinase family protein		Solyc07g045160.2.1	1,53
Fructose-1 6-bisphosphatase class 1		Solyc09g011810.2.1	3,16
Fructose-1 6-bisphosphatase class 1		Solyc04g071340.2.1	1,46
Fructose-1 6-bisphosphatase class 1		Solyc10g086730.1.1	1,35
Fructose-1 6-bisphosphatase class 1	Chloroplast sedoheptulose-1,7-bisphosphatase	Solyc05g052600.2.1	1,08
Fructose-bisphosphate aldolase		Solyc02g062340.2.1	3,23
Fructose-bisphosphate aldolase		Solyc01g110360.2.1	1,83
Fructose-bisphosphate aldolase		Solyc02g084440.2.1	1,17
**Chlorophyll breakdown**			
Senescence-inducible chloroplast stay-green protein 2	Green flesh protein; Senescence-inducible chloroplast stay-green protein 1	Solyc08g080090.2.1	1,02
**Cytochrome b6f complex and plastocyanin**			
Plastoquinol-plastocyanin reductase		Solyc01g109040.2.1	2,69
Plastocyanin	Plastocyanin	Solyc04g082010.1.1	1,56
Cytochrome b6-f complex iron-sulfur subunit		Solyc12g005630.1.1	1,39
**ATP synthases**			
ATP synthase gamma chain		Solyc02g080540.1.1	2,54
ATP synthase delta subunit		Solyc12g056830.1.1	1,43
ATP synthase F1 delta subunit		Solyc05g050500.1.1	1,27
ATP synthase subunit b&apos		Solyc06g066000.1.1	0,85
**NADP reductase**			
Ferredoxin--NADP reductase		Solyc02g062130.2.1	1,20
Ferredoxin--NADP reductase		Solyc02g083810.2.1	0,98

We also observed up-regulation of genes involved in chlorophyll metabolism. Six genes involved in chlorophyll biosynthesis and one gene involved in chlorophyll degradation (encoding Senescence-inducible chloroplast stay-green protein 2) were also found to be up-regulated. Genes involved in chlorophyll biosynthesis encoded proteins including: magnesium chelatase H subunit, magnesium-protoporphyrin IX methyltransferase, magnesium-protoporphyrin IX monomethyl ester, light dependent NADH:protochlorophyllide oxidoreductase 1, chlorophyllide a oxygenase and geranylgeranyl reductase. In this context, it was of particular interest to observe the up-regulation of the Golden 2-like transcription factor (*SlGLK2*) (log_2_ ratio of 1.5) (Additional file 3: Table S3)*.* This gene belongs to the *GARP* family of *MYB* transcription factors, and determines chlorophyll accumulation and distribution in developing fruit, thus influencing the entire photosynthetic process [25]. For chlorophyll degradation, we noted a slight up-regulation of the senescence-inducible chloroplast stay-green protein 1 (*SlSGR1*) in MYC fruit (log_2_ ratio of 1). Stay-green genes encode members of a family of chloroplast proteins, involved in the breakdown of chlorophyll–apoprotein complexes [26].

Genes involved in photorespiration (encoding serine hydroxymethyltransferase, hydroxypyruvate reductase, serine-glyoxylate aminotransferase, glycine decarboxylase and glycolate oxidase were also up-regulated (Additional file 3: Table S3) in MYC fruit, suggesting that the photorespiratory activity in the fruit was probably higher under the mycorrhizal conditions.

Taken as a whole, our transcript profiling data are consistent with numerous studies reporting the expression of genes involved in light and dark photosynthetic reactions, and photorespiration at turning and later ripening stages of the tomato fruit [13, 27–29]. Biochemical data showed a decrease in enzymatic activity of glycolate oxidase and hydroxypyruvate reductase from breaker to red ripe stages [27] while a time-course analysis of genome-wide gene expression [13] showed that the large majority of photosynthetic and respiratory genes identified in our experiment are down-regulated during the transition from green to red ripe fruit. Given that the nutritional status of the fruits from MYC and CONT plants was comparable, and all these genes were up-regulated in the MYC fruit, we hypothesize that mycorrhization may help extend the duration of the photosynthetic and photorespiratory activities in tomato fruit.

### Carbohydrate and cell wall metabolism

**Figure 3:**
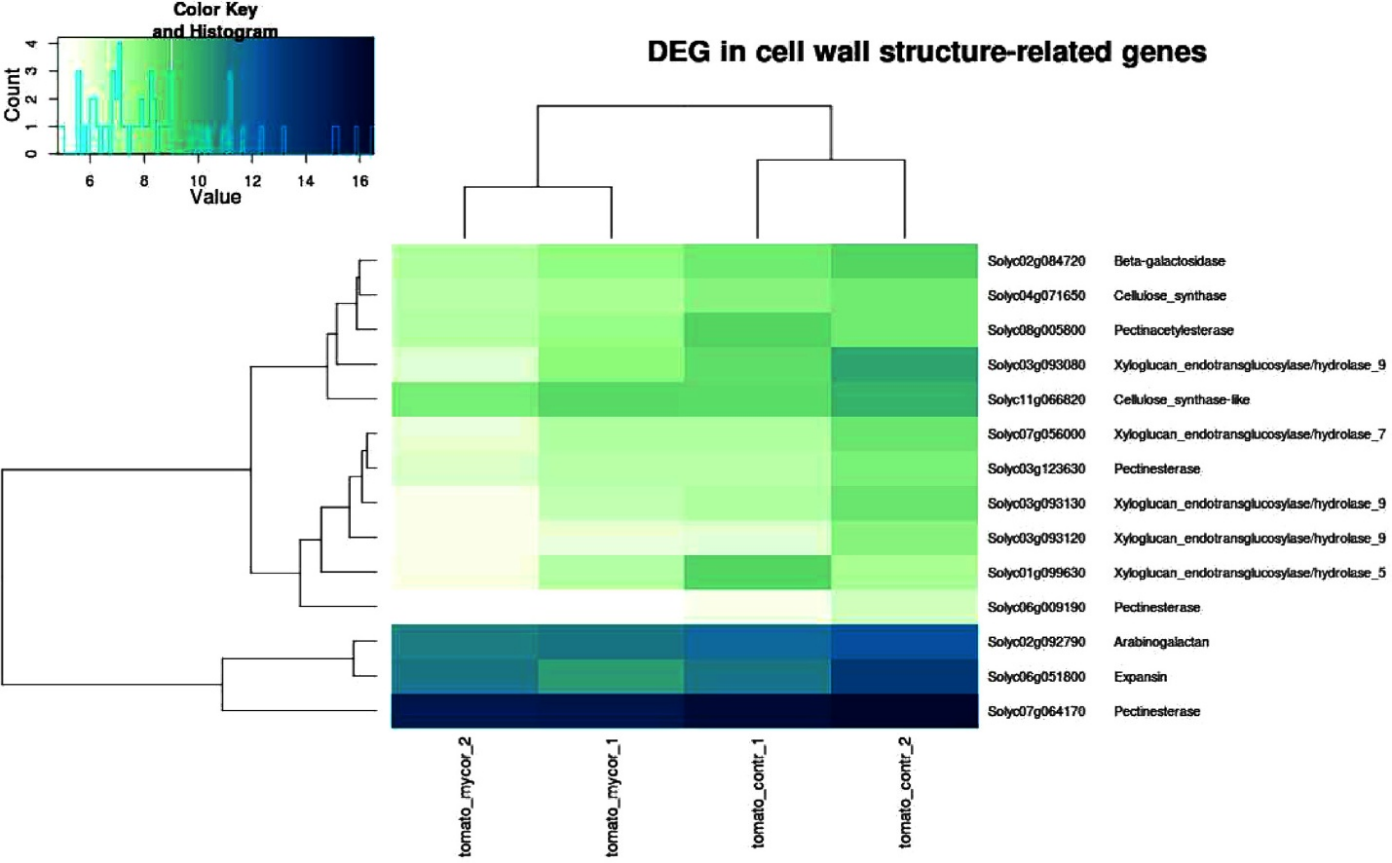
**Clustering and heatmap of expression values for DEGs in the cell wall structure-related gene class.** Light and dark blue indicate higher and lower expression values, respectively. Gene descriptions are as reported in Tomato Genome Consortium (2012). The heatmap was generated starting from DESeq normalized counts and further processed with custom scripts based on heatmap.2 function as available in the ‘gplots’ Bioconductor package and transformation function VST (DESEq package).

Tomato fruit ripening involves accumulation of sugars and breakdown of cell wall carbohydrates, resulting in fruit softening. We identified twenty differentially regulated genes belonging to the carbohydrate metabolic process group, eight that are up-regulated in MYC fruit and twelve that are down-regulated (Table 4). Almost all genes involved in cell-wall carbohydrate modification were down-regulated (Figure 3) and many of them (glycoside hydrolase family 28 protein, beta-1-3-glucanase, beta-galactosidase, endo-1-4-beta-xylanase and polygalacturonase 2) are known to be involved in the degradation of cell-wall integrity, inducing fruit softening during ripening [30–33]. In the same category, the 5-xyloglucan endotransglucosylase/hydrolases (*XTH*) which encode enzymes with xyloglucan endotransglucosylase (XET) or endohydrolase (XEH) activities were also down-regulated. The role of this gene family during tomato ripening has been extensively investigated [34–36] and *XTH* genes show different behaviors during ripening. In particular, *XTH1*, *XTH7*, *XTH12* and *XTH6* had their highest expression in the mature green stage and then decreased in the later stages.

**Table 4:** **Mycorrhiza-regulated genes involved in carbohydrate and cell-wall metabolism in turning tomato fruit**

Gene ID	ITAG2.3_descriptions	Description SL2.40	Log _2_ ratio MYC vs CONTR
**Carbohydrate metabolism**			
Solyc10g083290.1.1	Beta-fructofuranosidase insoluble isoenzyme 2	Cell-wall invertase	4,35
Solyc09g011810.2.1	Fructose-1 6-bisphosphatase class 1		3,16
Solyc02g082920.2.1	Endochitinase (Chitinase)	Acidic 26 kDa endochitinase	1,76
Solyc11g007990.1.1	Malate dehydrogenase	Malate dehydrogenase	1,66
Solyc04g071340.2.1	Fructose-1 6-bisphosphatase class 1		1,46
Solyc10g086730.1.1	Fructose-1 6-bisphosphatase class 1		1,35
Solyc05g052600.2.1	Fructose-1 6-bisphosphatase class 1	Chloroplast sedoheptulose-1,7-bisphosphatase	1,08
Solyc05g013810.2.1	Glycosyl hydrolase family 5 protein/cellulase		1,06
Solyc03g113030.2.1	Aldse 1-epimerase-like protein		-0,82
Solyc08g082170.2.1	Glycoside hydrolase family 28 protein		-0,83
Solyc04g016470.2.1	Beta-1 3-glucanase	Glucan endo-1,3-beta-D-glucosidase	-1,31
Solyc02g084720.2.1	Beta-galactosidase	Beta-galactosidase	-1,40
Solyc03g083910.2.1	Acid beta-fructofuranosidase	Acid beta-fructofuranosidase	-1,63
Solyc07g056000.2.1	Xyloglucan endotransglucosylase/hydrolase 7	Xyloglycan endo-transglycosylase	-1,70
Solyc03g093130.2.1	Xyloglucan endotransglucosylase/hydrolase 9	Xyloglucan endotransglucosylase-hydrolase XTH3	-2,28
Solyc01g111340.2.1	Endo-1 4-beta-xylanase		-2,33
Solyc01g099630.2.1	Xyloglucan endotransglucosylase/hydrolase 5	Probable xyloglucan endotransglucosylase/hydrolase 1	-2,37
Solyc03g093120.2.1	Xyloglucan endotransglucosylase/hydrolase 9		-2,49
Solyc05g005170.2.1	Polygalacturonase 2		-5,01
**Other cell wall genes**			
Solyc07g064170.2.1	Pectinesterase	Pectinesterase 1	-1,15
Solyc03g123630.2.1	Pectinesterase	Pectinesterase/pectinesterase inhibitor U1	-1,34
Solyc01g112000.2.1	Expansin-like protein	Expansin-like protein	-1,53
Solyc06g051800.2.1	Expansin	Expansin 1 protein	-1,62

Our data also show the down-regulation of other genes encoding cell wall associated proteins such as pectinesterases and expansins (Table 4). Expansins are involved in expansion of plant cells in growing tissues but were also identified in non-growing tissues [37]. One of the expansins identified in our experiment is *LeExp1* (Solyc06g051800.2.1) which was found to be specifically expressed in ripening fruit, with expression that was very weak at the green stage and increased with ripening [13]. In addition, *LeExp1* seemed to be directly regulated by ethylene at the transcriptional level [37]. The second class of cell-wall modifying proteins identified was the pectinesterases. Pectinesterases demethylate pectins and make them more susceptible to polygalacturonase action [38], but they may also reinforce cell-to-cell adhesion [39]. The down-regulated pectinesterase gene identified in our experiment (*PME 1.9*) (Solyc07g064170.2.1) was one of the five most highly-expressed genes among the DEGs (Table 2).

All together, the results related to the regulation of genes encoding cell wall modifying enzymes show that, with few exceptions, the genes involved in cell wall hydrolysis are repressed in the MYC fruit, suggesting that the molecular mechanisms that lead to softening are delayed in the MYC fruit.

The MYC fruit also showed down-regulation of an acid beta-fructofuranosidase (Solyc03g083910.2.1; log_2_ ratio: -1.635) coupled with a higher up-regulation of a cell-wall invertase (Solyc10g083290.1.1; log_2_ ratio: 4.35) (Table 4). BLAST searches of these two sequences showed that they are, respectively, the tomato vacuolar invertase *TIV1* and the cell-wall invertase (*LIN6*). These vacuolar and cell wall acid invertases, cleave the sucrose transported from source organs (leaves) into hexoses (glucose and fructose). Heterotrophic organs like fruit then use the hexoses as a direct source of carbon and energy [40]. The supply of sucrose to fruit and other sink tissues can limit their growth [41]. In carbohydrate supply to sink organs, sieve elements release sucrose into the apoplast where it undergoes an irreversible hydrolysis catalyzed by extracellular invertase and sucrose synthase [42]. The resulting hexoses are then transferred by hexose transporter(s) into sink cells. In tomato, extracellular invertases are encoded by *LIN5*, *LIN6*, *LIN7* and *LIN8*, which have highly tissue-specific expression, regulated by internal and external signals [42]. *TIV1* is expressed in red tomato fruit and *LIN6* is highly expressed in seedling roots, flowers and tumors, but not in fruit [42]. Our RNA-Seq data show very low expression of *LIN6* in CONT fruits (1.35 normalized read counts) but a much higher expression in MYC fruits (27.75 normalized read counts). Together, these data suggest that sucrose is actively mobilized in the MYC fruit, providing ready-to-use sugars for fruit development.

### Transport processes

**Figure 4:**
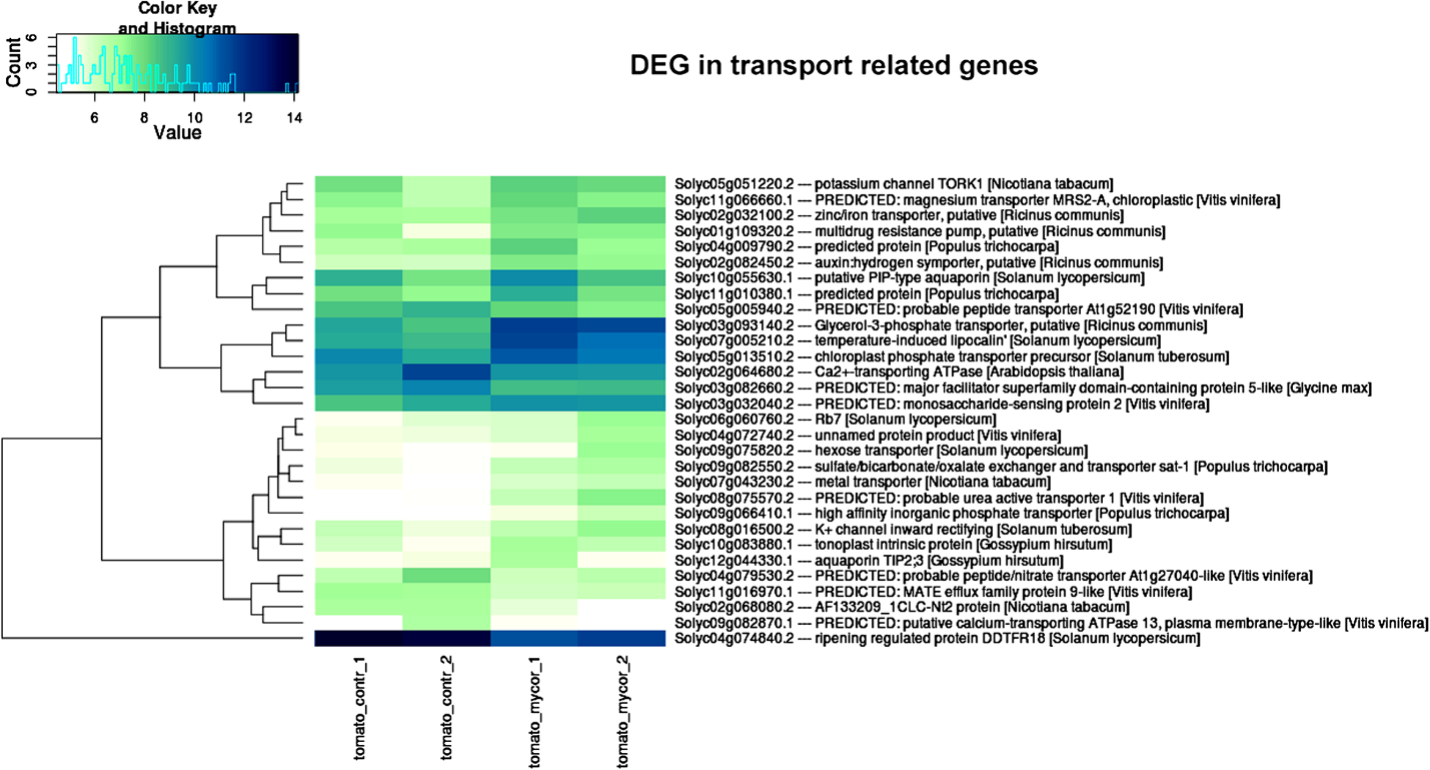
**Clustering and heatmap of expression values for DEGs in the transmembrane transport-related gene class.** Light and dark blue indicate higher and lower expression values, respectively. Descriptions as obtained from BLAST2GO annotation are shown. The heatmap was generated starting from DESeq normalized counts and further processed with custom scripts based on heatmap.2 function as available in the ‘gplots’ Bioconductor package and transformation function VST (DESEq package).

We identified 30 DEGs as involved in transport processes (Figure 4), and some of them are related to the DEGs involved in primary and carbohydrate metabolism. Among them, one of the most up-regulated genes in MYC fruit was a hexose transporter 2 (*LeHT2*) (Solyc09g075820.2.1; log_2_ ratio: 3.24). This gene shows the highest sequence similarity to a glucose/H + and low-affinity fructose/H + symporter that is expressed in tomato fruit storage parenchyma cells and strongly induced at fruit maturity [43, 44]. Hexose transporters transfer the hexoses hydrolyzed by cell wall invertases into sink cells. Therefore, they play an important role in sugar accumulation by fruit storage cells [45]. Indeed, a significant reduction (80–90%) in expression levels of three hexose transporter genes, *LeHT1*, *LeHT2*, *LeHT3*, by RNAi-mediated knockdown caused a 55% decrease in fruit hexose accumulation [44].

A tomato hexose transporter has been described as up-regulated after infection with Tomato yellow leaf curl virus and as co-regulated with a tomato lipocalin [46]. Lipocalins transport small hydrophobic molecules, and play important roles in various biological processes such as development and stress responses. Interestingly, a tomato lipocalin (Solyc07g005210.2.1) was activated in the MYC fruits, suggesting a network of interactions between transporters and responses to biotic stresses (see below).

We observed the up-regulation of one plasma membrane intrinsic protein and three tonoplast intrinsic protein aquaporins. Aquaporins function in water transport, and play pivotal roles in regulating water movements in fruit, which are crucial for fruit expansion and ripening [47]. Aquaporins are highly responsive to mycorrhization in legumes [48–52], although their exact role in the symbiosis is not clear [53] and tomato roots may also show a downregulation of aquaporins [6].

A urea transporter, characterized by a high up-regulation in the MYC fruit, showed 71% amino acid sequence similarity with an *Arabidopsis thaliana* sodium/urea cotransporter (DUR3). Interestingly, an allantoinase gene was found to be up-regulated in MYC fruit of the Micro-Tom variety, suggesting that the metabolism of urea precursors is also activated [8].

In accordance with the prominent function of transport in mycorrhizal roots, the membrane transporters functional category was found to be one of the most-regulated categories in roots colonized by AM fungi [50, 51, 54].

Our experiments identified two up-regulated genes involved in phosphate transport. The inorganic phosphate transporter (Solyc09g066410.1) has homology to both *LePT1* and *LePT3* phosphate transporters. Unlike *LePT1,* which is constitutively expressed [4], Solyc09g066410.1 seems to be induced by mycorrhization as it showed no basal expression in the fruit of non-inoculated plants. The second identified phosphate transporter (Solyc05g013510.2) shows the highest similarity with a chloroplastic inorganic phosphate transporter from *S. tuberosum*; this gene was highly expressed in young, rapidly growing tissues and may have a role in cell wall metabolism [55]. These data provide a good mechanistic basis for the observed fruit P content, which reaches comparable values in the two MYC and CONT conditions. This suggests that the mycorrhizal- dependent P uptake is as efficient as the P uptake from fertilization. Similarly, two sulphate-related transporters (High affinity sulphate transporter, see Additional file 3: Table S3) were more strongly expressed in the fruits from mycorrhizal plants, consistent with the comparable S content.

A solute carrier protein, probably acting as a nitrate transporter, was found to be down-regulated in our dataset. Nitrate transporters were found to be either up-or down-regulated in *Medicago* mycorrhizal roots, depending on the specific gene considered [48, 51, 56]. In tomato, colonization by *F. mosseae* specifically enhanced the expression of the nitrate transporter *LeNRT2;3* without affecting the expression of other isoforms [57].

In conclusion, in addition to the expression of genes that function in fruit development, the presence of the AM fungus activates genes involved in nutrient and water transport activities, which are strictly related to the known functional markers of mycorrhizal roots.

### Regulation of hormonal pathway genes

Tomato fruit development and ripening is stringently regulated by hormone dynamics [22]. In roots, the hormonal balance modulates mycorrhization and vice-versa [7, 58–60]. In addition, tomato genes involved in hormonal metabolism show a systemic response to the AM fungus [6]. We therefore expected hormone pathway genes to be differentially regulated in fruit; indeed, 11 genes were found to be involved in gibberellin, abscisic acid (ABA) and ethylene metabolism (Additional file 3: Table S3). A Gibberellin 2-oxidase 1 involved in the degradation of the active gibberellic acid (GA), and two Gibberellin regulated proteins, *GAST1* (gibberellic acid stimulated transcript) and *RSI-1* (Root system inducible-1), were found to be down-regulated in the fruit of MYC plants. Both *GAST1* and *RSI-1* belong to the GASA (*GA-stimulated Arabidopsis*) gene family, which includes many hormone-responsive genes involved in pathogen responses as well as in different aspects of plant development [61]. Two genes involved in ABA metabolism were found to be up-regulated in MYC fruit: the first one is zeaxanthin epoxidase, which catalyzes the conversion of zeaxanthin to violaxanthin [62], an ABA precursor [63]. The second gene is the ABA 8′-hydroxylase *CYP707A2*, which plays a role in ABA catabolism and is activated by the presence of ABA [64].

Six ethylene responsive factors were down-regulated in the MYC fruit (Additional file 3: Table S3): Ethylene response factor 1 (*ERF1*), a probable ethylene responsive transcription factor 2b, the C-repeat-binding factor 3 (*CBF3*), a probable ethylene-responsive transcription factor 1, the ethylene-responsive transcription factor 10 and the ethylene-responsive late embryogenesis-like protein. The *ERF*s are secondary ethylene activated factors, which increase plant tolerance to stress by modulating expression of downstream stress-responsive genes [65]. *ERF1* is a key element in the response to different necrotrophic pathogens [66]. Interestingly, in *Medicago* the transcription factor *ERF19* is down-regulated by the perception of an effector molecule secreted by an AM fungus [67]. However its most closely related homologue in tomato does not show differential expression during fruit ripening.

Overall, these data demonstrate the dynamic expression of genes involved in hormone metabolism: on the one hand, they mirror the transcriptional profile of fruit ripening markers (ethylene-responsive genes) and related pathways; on the other, some of them mirror the known role of hormone networks in AM establishment. Indeed, a positive correlation between ABA and mycorrhization in roots was reported [58] and ABA was found to be necessary for complete and fully functional arbuscule development and for successful colonization of the plant root [58]. By contrast, numerous studies have shown a reciprocal negative relationship between ethylene and mycorrhization [7, 59]. However, the role of hormone networks in AM establishment remains far from fully understood [7, 60].

### Terpenoid metabolism

Terpenoids include phytohormones (abscisic acid, gibberellins, cytokinins, and brassinosteroids), photosynthetic pigments (phytol and carotenoids), and electron carriers (ubiquinone) [68]. However, most terpenoids are specialized secondary metabolites that function as defensive compounds accumulating upon pathogen infection (i.e. phytoalexins) [69] or attracting predators/parasitoids of the attacking insect [70]. In tomato, terpenoids are present in large quantities in the glandular trichomes on leaves, stems, young fruits, and flowers [71–74].

Three genes belonging to the terpene synthase (*TPS*) family, *TPS31*, *TPS32* and *TPS33*, were induced in the tomato fruit by mycorrhization. Although only a few genes were affected, these three genes were among the ten most up-regulated by mycorrhization (Additional file 3: Table S3) and *TPS31* showed no basal expression in CONT tomato fruits. *TPS31*, *TPS32* and *TPS33* belong to the sesquiterpenoid phytoalexin biosynthesis pathway (Additional file 2: Figure S4), leading to the production of the phytoalexin rishitin, well known for its antimicrobial properties [69]. Accumulation of phytoalexins has been suggested as a defense mechanism in mycorrhizal plant roots [75–77]. By contrast, genes involved in carotenoid synthesis did not show any change in their transcriptomic profiles (Additional file 2: Figure S5). These data were confirmed by a biochemical analysis of these metabolites, which showed no statistically significant differences between the two sets of fruits (Additional file 1: Table S4), with the exception of general reducing activity, which was higher in the MYC fruits.

### Defense genes

Plants activate a wide range of mechanisms to defend themselves against external attacks [78]. Such responses are under the control of a finely tuned hormonal network, and can spread from the infection site to a systemic level. In our study, four out of the five up-regulated genes were pathogenesis related proteins (PRs) three *PR10s*, and one *PR1*. PRs specifically accumulate in plants in response to pathogen infection and act in development of systemic acquired resistance against further infections. PR regulation in response to mycorrhization appears to be complex; López-Ráez and colleagues [7] reported the induction of *PR10* in tomato roots inoculated with *Funneliformis mosseae* and *Rhizophagus intraradices*. By contrast, some PRs were down-regulated in plant shoots following mycorrhization [6, 49] and induced only after a pathogen attack, as a result of the mycorrhizal priming effect [79]. In our experiment, we observed that mycorrhization *per se* induced the up-regulation of *PRs* in tomato fruit (Additional file 3: Table S3), as already observed for *PR1* in rice leaves [80]. Another gene (an acidic endochitinase) up-regulated in the fruit from MYC plants can be grouped in this category, as chitinase induction results in the activation of a mycorrhiza-mediated defense response to worms in grapevine roots [81] as well as in mycorrhizal rice leaves [80].

Finally, a gene coding for a plant defensin containing a gamma-thionin domain was found to be down-regulated. Defensins are part of the front line of the plant immune system, inhibiting the growth of bacteria and fungi. Strikingly, three defensins were highly induced in AM-colonized *Medicago* roots, and their expression was strongly up-regulated in arbuscule-containing cells [51].

A number of papers have reported expression of homologues of these tomato genes in roots, shoot and leaves of mycorrhizal plants, opening the question whether AM fungi influence host immunity by modulating defense responses [82]. Our data suggest that some of these responses also remain active at the systemic level. In conclusion, it seems that a set of defense-related genes –already identified in mycorrhizal plants – are differentially expressed in tomato fruit at the turning stage, suggesting that they could take part in the so-called priming reaction elicited by mycorrhizal fungi and are also maintained at systemic level in the fruit.

### Time course analysis and validation of selected DEGs

**Figure 5:**
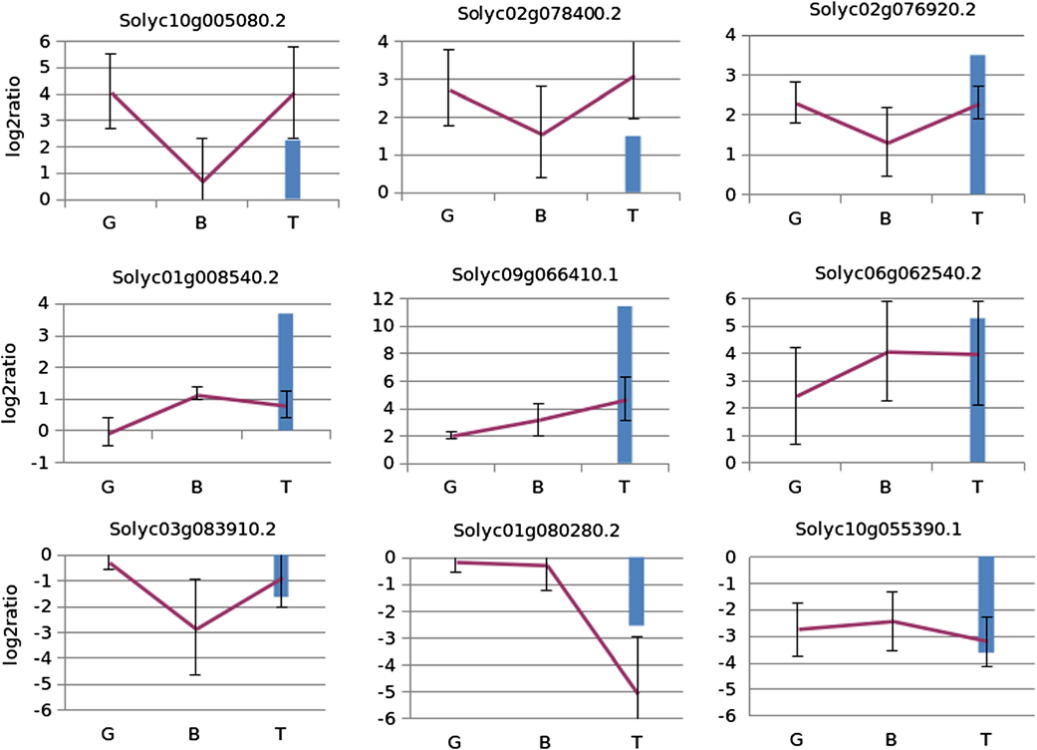
**q-PCR time course analysis of gene expression in fruits of different stages.** Relative gene expression in mature green (G), breaker (B) and turning (T) stage fruits, of nine selected differentially expressed genes (red line). The blue bar indicates the log2 fold change obtained at the turning stage in the RNAseq experiment. Identities of genes are as follows: Solyc01g008540.2.1 (Cinnamoyl CoA reductase-like protein); Solyc02g078400.2 (Allantoinase); Solyc02g076920.2.1 (Transcription factor MYC2); Solyc10g005080.2.1 (Late elongated hypocotyl and circadian clock associated-1-like); Solyc09g066410.1.1 (Inorganic phosphate transporter); Solyc06g062540.2.1 (Putative acid phosphatase); Solyc03g083910.2.1 (Acid beta-fructofuranosidase); Solyc01g080280.2.1 (Glutamine synthetase); Solyc10g055390.1 (Nodulin family protein).

To validate the RNA-Seq data and to check whether the effect of mycorrhization on gene expression maintained the same trend (up-or down-regulation) at different stages of ripening, we used quantitative real-time PCR (q-PCR) to examine the pattern of gene expression of nine selected DEGs at three fruit ripening stages: mature green, breaker and turning. We also tested a group of non-regulated genes involved in carotenoid metabolism: 1-deoxy-D-xylulose-5-phosphate synthase (*DXS*), 4-hydroxy- 3-methylbut-2-en-1-yl diphosphate reductase (*HDR*), phytoene synthase (*PSY*), carotenoid cleavage dioxygenase (*CCD*), and lipoxygenase (*LOX*). The trend of up- or down-regulation observed in the RNA-Seq experiment was validated for all the genes at the turning stage, and statistically significant changes were observed for 7 out of 9 genes in at least one time point (Figure 5; Additional file 2: Figure S6). None of the carotenoid metabolism genes turned out to be regulated by mycorrhization in q-PCR, according to the RNA-seq results (Additional file 2: Figure S5). These results verify the reproducibility and reliability of the RNA-Seq technique and suggest that the regulation of the selected genes by mycorrhization is not restricted to the turning stage at which the RNA-Seq was performed. Mycorrhization often exerted a more intense effect at other ripening stages, when a peak in gene expression was observed (Figure 5). The values were, in most cases, higher in RNA-Seq than in q-PCR experiments; this was mainly observed for genes with low fold-change levels. The time course q-PCR analysis allowed us to validate the changes in expression for genes with particular importance. For instance, the statistically significant up-regulation of *IPT* in all the three ripening stages allowed us to hypothesize that this gene may be a mycorrhiza-induced transporter, like other tomato phosphate transporters already found to be activated by mycorrhization in the roots [5, 83]. Our analysis indicates that *IPT* could be a good candidate to be used as a fruit marker for mycorrhization.

## Discussion

**Figure 6:**
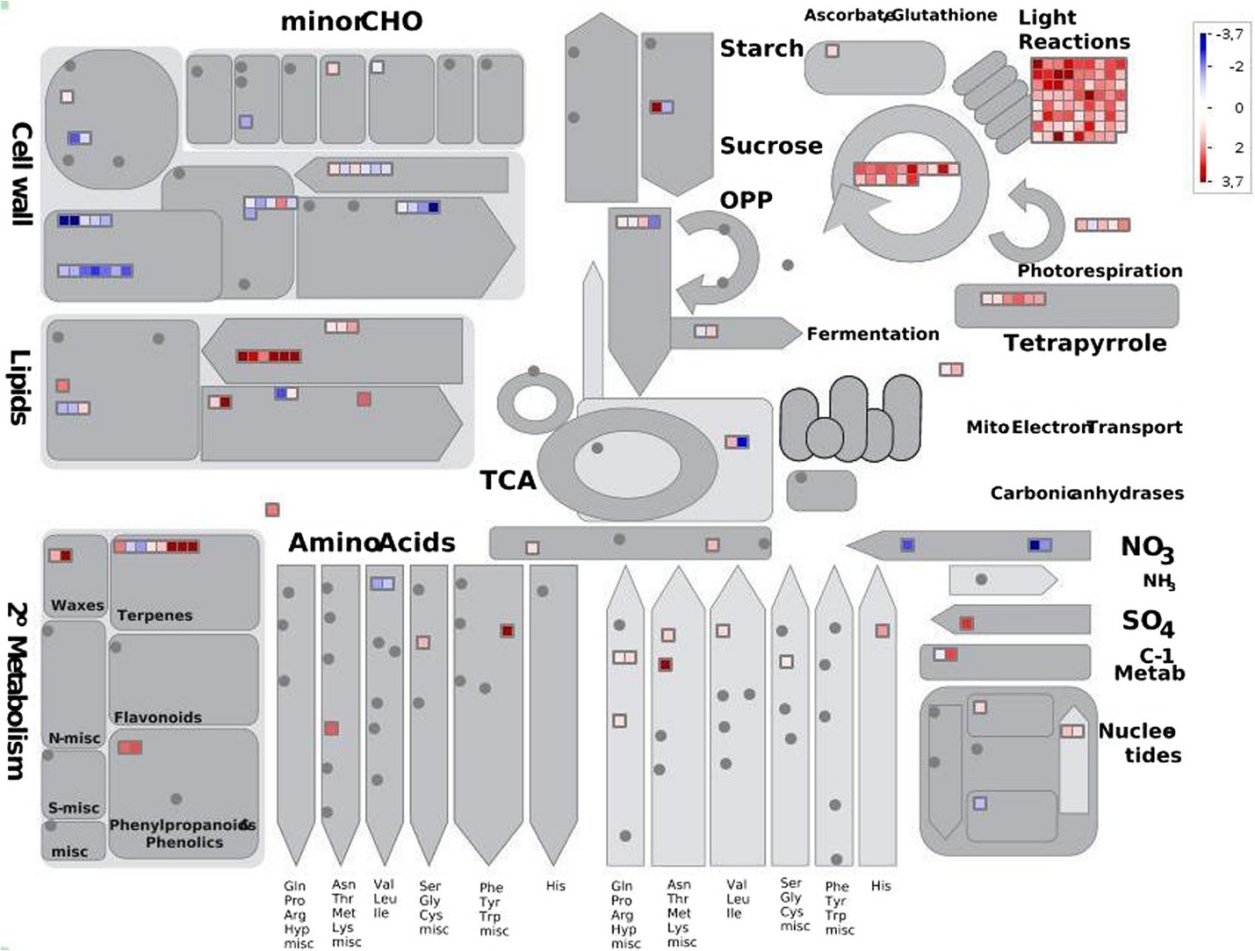
**Overview of the effect of mycorrhization-induced expression changes on metabolism of tomato fruits.** MapMan software (Metabolism_overview panel) was used to provide a snapshot of modulated genes over the main metabolic pathways. DEGs were binned to MapMan functional categories and Log_2_ fold changes values are represented. Up-regulated and down-regulated transcripts are shown in red and blue, respectively.

Our genome-wide RNA-Seq analysis of tomato fruits from mycorrhizal plants demonstrated that the presence of the mycorrhizal fungus in the roots influences genes involved in some processes strictly related to the ripening of the fleshy tomato fruit. Our strategy to compare mycorrhizal plants with fertilized plants allowed us to uncouple the nutritional effects from the presence of AM fungi, which improve plant nutrition [20]. In this way we avoided the stress conditions that are characteristic of control non-fertilized plants, as plants respond to nutrient deficiency with important changes in root and shoot architecture [84]. We cannot exclude that the different fertilization treatment may have affected the transcriptomic profile; however, our data strongly suggest that, in fruits, AM symbiosis affects a core set of genes that have already been identified as sensitive to the presence of AM fungi (i.e. nutrient transporters). Along the same line, AM symbiosis also affects transcripts involved in major fruit pathways including photosynthesis and photorespiration as well as amino acid and cell wall metabolism. Figure 6 shows an overview of the major expression changes that we interpreted as caused by mycorrhization, as annotated by MapMan software [85]. The fruits of mycorrhizal plants show transcriptomic signatures characteristic of a climacteric fleshy fruit, and of the mycorrhizal status of its roots. Thus, even if biological conclusions based on a transcriptome analysis remain speculative, the obtained results, coupled with information on fruit nutrient contents, allow us to discuss some hypotheses on the mechanisms by which AM fungi might influence tomato fruit.

### From roots to fruits: sharing similar transcription profiles

The bulk of the data available on gene expression in mycorrhizal conditions relates to tomato roots [54] as the physical site of mycorrhizal interaction and functioning. The fruit possesses a unique metabolism, leading to the production of specific metabolites, including pigments, flavor, and aroma compounds. It is thus particularly interesting, but at the same time tricky, to draw a parallel between the functional meaning of gene regulation in roots and what we observed for the first time in the fruit transcriptome of mycorrhizal tomato plants.

Out of the 712 regulated genes in the MYC fruit, 5 genes have homologues that have already been reported as activated in mycorrhizal plants; these include a TIP-aquaporin expressed in tomato leaves [86] a pathogenesis-related protein 10 [7] expressed in roots, and a probable root mycorrhiza-induced inorganic phosphate transporter [5]. Our RNA-Seq analysis also allowed us to validate the changes in expression of some genes identified in our previous microarray experiment on Micro-Tom fruit as regulated by mycorrhization [8]. For example, we confirmed the up-regulation of the allantoinase gene and the down-regulation of the glycolate oxidase 2 gene [8].

A huge number of transcriptomic investigations have shown the extensive effects of AM colonization on host metabolism [51, 54] and identified a core set of genes that respond to mycorrhization for all tested plants, fungi, and organs. Our global transcriptome profiling, which analyzed the tomato fruit transcriptome *via* RNA-Seq, has identified two gene categories where the systemic effect of the mycorrhization is of particular relevance: the transporters and the defense-related responses.

The up-regulation of transporter genes illustrates the specific lifestyle of mycorrhizae; AM fungi support the plant, improving its mineral nutrition and receiving fixed carbon. The regulation of transporter-coding genes in organs other than the roots strongly suggests that this function is also highly responsive to mycorrhization at a systemic level. This leads to the major functional result of our analysis: the transcriptomic signature and the mineral contents of the fruits demonstrate that the AM fungus improves plant and fruit nutrition as efficiently as heavy fertilization. It would be of interest to understand whether in field conditions, mycorrhization could confer the same advantages as chemical fertilization, in terms of fruit quality and/or energy consumption.

Similarly, several studies have examined the effects of mycorrhization on plant defense mechanisms. Recent research suggests that mycorrhizal establishment stimulates priming of the plant’s innate immune system rather than the induction of specific defense mechanisms [82, 87]. Mycorrhizal plants seem to react more promptly to pathogen attack, eventually showing enhanced resistance [80, 88]. Our results on the activation of PR proteins, homologs of others already reported as activated in AM roots [7], show that in the MYC fruit, more complex regulation takes place, as some of these genes were down-regulated in shoots [6, 49] while others were up-regulated [80].

### The AM fungus in the root affects the transcriptomic profile of genes involved in fruit photosynthesis, photorespiration and sink strength

Developing tomato fruits conduct photosynthesis and contribute up to 20% of the fruit’s photosynthate, as shown by their expression of proteins involved in light-harvesting, electron transfer and CO_2_ fixation [89]. During ripening, tomato fruits shift from partially photosynthetic to truly heterotrophic metabolism, as also mirrored by a decrease of many photosynthetic proteins together with those involved in chlorophyll biosynthesis [29]. However, notwithstanding the wealth of data, the role of fruit photosynthesis remains not fully understood [90]. Our transcriptomic analysis adds a novel piece of information to this emerging understanding, showing that mycorrhization might affect fruit photosynthesis and photorespiration. This transcriptional influence seems to be independent of the nutritional level, since mycorrhizal and control fertilized plants had similar mineral and nutrient contents in their fruits (Additional file 1: Table S1).

Here we found that tomato fruits of mycorrhizal plants sampled at the turning stage present an important up-regulation of photosynthetic genes, which are usually activated in immature green fruits [91]. Also, in MYC fruits photorespiration may be activated via the increased expression of a core set of genes (Additional file 2: Figure S2). AM fungi stimulate photosynthesis in leaves of different plants [92, 93]. Interestingly, strigolactones, which act as positive regulators of AM symbiosis, also up-regulate light-harvesting genes in the same tomato organs [94]. It has been proposed that mycorrhization causes an improvement of plant water status, increasing hydraulic conductance and affecting stomata and leaf expansion [95, 96]. Other investigations suggested that the AM fungi, which depend on their host for carbon, increase root sink strength and enhance leaf photosynthetic activity to compensate for the ‘cost’ imposed upon the plant C economy by the fungus [97, 98].

On the basis of our RNA-Seq profiling, we hypothesize that similar events also take place in the fruit, where the increased sink strength is confirmed by the activation of other metabolic pathways. The up-regulation of *LIN6* in our MYC fruit is particularly intriguing as the expression of this gene has been reported to be high in roots, flowers and tumors and was almost absent in fruit [42]. Indeed, in our experiment, *LIN6* was very weakly expressed in the CONT fruit.

It seems therefore that mycorrhization positively influences the expression of genes of the metabolic pathways related to carbon assimilation and respiration. In this context, the up-regulation of the SlGLK2 transcription factor [25] could provide novel and unexpected insights. SlGLK2 is encoded by the uniform ripening locus (*u*) and determines the intensity and pattern of chlorophyll distribution in the unripe fruit. The dominant *U* allele leads to fruit with dark green shoulders at the stem end adjacent to the pedicel, and *u*/*u* fruit are uniformly light green and were therefore favored by the breeders. However, in these fruits selection has led to suboptimal chloroplast development, and consequently, to decreased ripe fruit sugar and lycopene levels. It seems that in some way mycorrhization rescues the levels of *SlGLK2* mRNA to higher levels, also probably controlling the downstream photosynthesis-related genes.

### Could mycorrhization extend tomato shelf life?

The ripening of climacteric fruits such as tomato is characterized by a large burst of respiration known as the ‘climacteric rise’ at the onset of ripening. The climacteric rise is coupled with increased ethylene biosynthesis [39], which is thought to coordinate numerous biochemical and physiological processes necessary for normal ripening such as softening of the cell wall, accumulation of pigment and increases in flavor and aroma [14]. Cell wall softening, an important process influencing fruit quality and principally shelf life, involves a coordinated decline in the activity of cell-wall biosynthetic enzymes and an increase in the activity of cell wall hydrolytic enzymes, like polygalacturonase [99].

Availability of the high-quality, annotated tomato genome sequence [13] has allowed an extension of our understanding of ripening, identifying more than 40 genes involved in fruit cell wall architecture. The regulation of cell wall related genes strictly relates phenotypic features, including fruit firmness, and shelf life. Our transcriptomic data consistently demonstrate that mycorrhization may lead to a down-regulation of cell wall hydrolytic enzymes. Similar results were already reported in a previous, more limited study [8] of the Micro-Tom variety.

All these data, together with the up-regulation of the *SlGLK2* transcription factor, the up-regulation of photosynthesis-related genes, the increased chlorophyll synthesis and the down-regulation of ethylene responsive genes, indicate that mycorrhization may lead to fruits with a potentially longer shelf-life through the activation of multiple pathways.

## Conclusions

The extensive transcriptomic analysis we performed, comparing tomato fruits from mycorrhizal plants with fruits from fertilized plants, demonstrates that mycorrhization may have important systemic effects. By improving the nutritional status of the whole plant, mycorrhization leads to a mineral content in the fruits that is comparable to the mineral content of fruits from highly fertilized plants. By affecting the source-sink relationships of the whole plant, mycorrhization also has an impact on some of the features of fleshy fruits. This novel transcriptome survey thus shows biological intersections between roots and fruits, and will provide a platform for future, in-depth studies. The data we present here will allow study of the complex process of tomato fruit development under the usual cultivation conditions, like those occurring in greenhouses where the plants are fertilized, the soil is not sterile, and the complex tomato microbiome, also including AM fungi, affect plant performance.

## Methods

### Plant materials and growth conditions

*Solanum lycopersicum* cv. Moneymaker tomato plants were grown in the greenhouse from March through December under natural light, which provided a photoperiod ranging from 9 h to 15 h, temperature from 16°C to 30°C and relative humidity from 55% to 80%. The seedlings were grown in pots with sterile quartz sand (80%) and sterile pumice (20%) for non-inoculated plants or with a mixture of sand (55%), sterile pumice (20%) and *Funneliformis mosseae* (formerly *Glomus mosseae*) inoculum (25%) Gerd. & Trappe (BEG 12) purchased from Agrauxine (Bretagne, France, http://www.agrauxine.fr) for inoculated plants. Forty-five plants divided into three groups of fifteen plants according to their growth conditions were considered: plants grown with *F. mosseae* inoculum (MYC) or without inoculum were watered, once a week, with a modified Long-Ashton solution containing a low phosphorus and nitrogen concentration (3.2 μM P, 1 mM N, 1.75 mM S, 1 mM K) [100]. The fertilized control plants (CONT) were grown without inoculum and watered, once a week, with a commercial fertilizer solution (3.8 mM P, 12.9 mM N, 6.6 mM S, 3.8 mM K) (Asso di fiori, CIFO S.p.A). Phenological features were monitored during the experiments, as summarized in Additional file 1: Table S2. At the end of the experiment, roots from MYC and CONT plants were cut and fungal colonization was assessed according to the Trouvelot system [101] using MYCOCALC software.

### Illumina sequencing

For the RNA-Seq experiment, fruits were harvested from the CONT and MYC plants when the fruits reached the turning stage. RNA was extracted using the ‘pine tree-method’ [102] with the addition of 1% PVPP to the extraction buffer. For each growth condition, we used two biological replicates, each containing the pooled RNA from three fruits. Ten micrograms of each RNA sample was sent to Fasteris Life Science Co. (Geneva, Switzerland) where the libraries were produced and sequenced using the Illumina Genome Analyzer (Solexa). The four libraries were indexed and single-end multiplexed sequencing was performed using 100 bp length reads.

### Time course quantitative Real-Time PCR validation

Quantitative Real-Time PCR, was used to measure the expression of nine genes shown to be differentially regulated by RNA-Seq, as well as of five carotenoid related genes. Three fruit ripening stages were considered: mature green, breaker and turning. For each stage, three biological replicates were used. RNA extraction, primer design, quantitative real time PCR experiment and data analysis were carried out as described in Salvioli et al. [8]. The primer names and corresponding sequences are listed in Additional file 1: Table S5.

### Bioinformatic methods

#### Mapping of Illumina reads

Raw fastQ files were checked for contaminants and low-quality bases and contaminants were removed with the cutadapt software [103]. The spliced read mapper TopHat version 1.4.1 [104] was used to map reads to Tomato SL2.40 (ITAG2.3) obtained from the plant repository. A minimum and maximum intron length of 40 and 50,000 bp were used, respectively. Read counts were collected with HTSeq version 0.5.3 (http://www-huber.embl.de/users/anders/HTSeq) in the single end and ‘union’ mode using *Solanum lycopersicum* MSU SL2.40-16 GTF file as obtained from the ensembl plant repository.

#### DEG calling

The DESeq Bioconductor package version 1.10.1 [105] was used to call differentially expressed genes. DESeq implements a model based on negative binomial distribution; this model was developed with special attention to coping with biological variance and was run under R release 2.15.2. The cutoff for considering a gene expressed was set to 0.1 RPKM (Reads per Kilobase per Million). The False Discovery Rate (FDR) threshold was set to 0.1 and gene dispersion values were calculated by fitting a curve according to the DESeq “fit-only” mode.

#### GO enrichment analyses

GO enrichment analyses were conducted with the goseq bioconductor package version 1.10.0. As tomato databases are not yet covered by goseq built-in databases, transcripts lengths were retrieved with BiomaRt queries (*Solanum lycopersicum* SL2.40). An FDR cutoff of 0.1 was used for goseq GO enrichments. Gene ontology terms for CC, MF and BP were similarly retrieved with BiomaRT queries.

#### Other bioinformatic techniques

Pie charts for the Distribution of GOSLIM BP terms were generated via BLAST2GO software using SL2.40 annotations. MapMan figures were obtained by first running the mercator tool (http://mapman.gabipd.org/web/guest/mercator) with default parameters to assign MapMan bins to tomato transcripts. Log_2_ fold changes as obtained from DESeq output were used as MapMan input to represent expression changes. For clustering and heatmaps of expression values, DESeq-normalized data were prior transformed with the Variance-stabilizing transformation (VST) as described by Anders and Huber [105]. Unless otherwise stated, further graphical outputs were generated with custom R and Python scripts. Custom annotations/descriptions were used as specified in the text and figures and were obtained with BLAST2GO on tomato SL2.40 cDNAs run with the following annotation parameters: E-value hit filter 1.E-10, Annotation cutoff 55, Go weight 5, Hsp-Hit coverage cutoff 20. Further complementary descriptions (ITAG2.3_descriptions) were obtained from ftp://ftp.solgenomics.net/tomato_genome/annotation/ITAG2.3_release/.

### Fruit mineral content measurements

About 5 g of frozen tomato fruit pericarp from MYC and CONT plants were dried at 70°C for 48 hours, and digested in 2 ml 6 M HNO_3_ at 90°C for one hour. The digestion product was diluted to 6 ml with milliQ water and filtered. The P, S and K contents were determined in the final solution using Inductively Coupled Plasma Atomic Emission Spectrometry (ICP-AES) performed using a Liberty 100 Varian apparatus equipped with a V-Groove nebulizer and a Czerny–Turner monochromator (Department of Mineralogical and Petrological Science, University of Turin). The same treatments were applied to the control solution, which contained no sample. Quality control was based on the use of internal control samples and certified samples (Astasol-Mix from Analytica Ltd., Prague, Czech Rep.). C and N content was measured on fruit powder with a CHNS-O analyzer (Thermo Electron Corporation).

### Biochemical analyses for nutraceutical values

#### Fruit extraction

About 6 g of unfrozen fruit material were extracted on a magnetic stirrer for 15 min in 30 ml volume of a methanol, distilled water and formic acid (80:20:0.5) mixture. The extracts were double-filtered, first on blotting paper with a Büchner funnel connected to a vacuum pump and then with a 0.45 nm cellulose membrane. The total antioxidant activity and the phenolic content of the methanolic fruit extracts were immediately assayed. All spectrophotometric measurements were performed in glass cuvettes using a T80+ UV/VIS spectrometer (PG Instruments Ltd.).

#### Total antioxidant activity

The total antioxidant activity of the tomato fruit extracts was measured by testing the radical scavenging capacity (DPPH test) and the reducing power (FRAP assay), according to the methods described by Tenore et al. [106].

#### Total Phenolic content determination

The total phenolic content was analyzed spectrophotometrically using the modified Folin–Ciocalteu method [107, 108]. 200 μl of the fruit extract from MYC and CONT plants was mixed with 100 μl of Folin-Ciocalteu’s phenol reagent (Sigma) in a glass test tube. Successively, 5 ml of 7% sodium carbonate in distilled water was added to the tube and the mixture was kept in the dark at room temperature for 2 h. Absorbance was then measured at 760 nm and results were expressed as mg Gallic acid equivalents (GAE) per 100 g of tomato fresh weight. Gallic acid (Sigma) was used to calculate the standard curve at 0.025, 0.05, 0.1 and 0.15 mg/100 μl.

#### Lycopene and β-Carotene

Quantification of Lycopene and β-Carotene was performed spectrophotometrically according to the method described by Nagata and Yamashita [109]. One g of unfrozen fruit tissues from MYC and CONT plants was homogenized and extracted in 16 ml of an acetone/n-hexane 40:60 solution for 15 min on a magnetic stirrer. Extracts were then filtered using a 0.45 nm cellulose membrane and absorbance at 663, 645, 505 and 453 nm was measured. Contents of β-Carotene and Lycopene were calculated according to the following equations: Lycopene (mg/100 ml) = - 0.0458 A663 + 0.3jou72 A505 – 0.0806 A453; β-Carotene (mg/100 ml) = 0.216 A663 – 0.304 A505 + 0.452 A453. Results were finally expressed as mg/100 g fm.

### Statistical analysis

For all the biochemical and nutraceutical measurements, five fruits were harvested for each MYC and CONT condition and originating from different plants. Values are expressed as mean ± standard deviation. Data were analyzed with a one-way ANOVA with Tukey post-hoc or the non parametric test Kruskal-Wallis accepting significant differences at p < 0.05. Significantly different values are highlighted in bold. All statistical analysis were performed with SYSTAT 10 statistical software.

### Availability of supporting data

Supporting sequence data are available in the ArrayExpress database (http://www.ebi.ac.uk/arrayexpress) under Accession number: E-MTAB-2276.

## Electronic supplementary material

Additional file 1: Table S1: Mineral content (C, N, K, P, S) of fully ripe CONTROL and MYC tomato fruit. Values are means ± SD (n = 5). DW, dry weight. Data highlighted in bold are significantly different (Non parametric test Kruskal-Wallis p < 0.05). **Table S2.** Phenology and fruit yield of CONT and MYC plants: length of the vegetative period (expressed in days after transplanting, DAT), number of days from flowering to the first fruit, fruiting period length, average fruit number per plant, total fruit production, average fruit weight and total fruit yield. Data are expressed as mean ± standard deviation, n = 10. Statistically significant data are highlighted in bold according to the non-parametric Kruskal-Wallis test (p < 0.05). **Table S4.** Biochemical and nutraceutical parameters measured in fully ripe CONTROL and MYC tomato fruits. Data highlighted in bold are significantly different (one-way ANOVA with Tukey’s posthoc test, p < 0.05). **Table S5.** Genes and corresponding primers used in real-time RT-PCR. (PDF 83 KB)

Additional file 2: Figure S1: Mycorrhization parameters of tomato roots inoculated with the AM fungus *Funneliformis mosseae*. F%: colonization frequency; M%: mycorrhizal colonization intensity; A%: arbuscule abundance; a%: arbuscule abundance in the mycorrhized portion. Mycorrhization parameters were determined according to Trouvelot method (1986). Values are means ± SD (n=5). For each plant, 100 cm of root were measured. **Figure S2.** Classification of tomato fruit expressed genes. All expressed genes (cutoff RPKM>0.1) are represented. The number of transcripts assigned to tomato biological process (GOSLIM plant mapping) is shown in parentheses. **Figure S3.** Transcriptional changes induced by mycorrhization in tomato fruits. Normalized expression mean values are plotted versus log2 fold changes and called DEGs (FDR<0.1) are plotted in red. Blue horizontal lines indicate a two-fold change in expression values for up- and down-regulated genes. **Figure S4.** Sesquiterpenoid phytoalexin biosynthesis pathway. **Figure S5.** q-PCR data for five selected genes involved in carotenoid metabolism on fruits from mycorrhizal and control plants. The y axis represents ∆CT value (Ct gene-Ct ubiquitin). Data are mean ± SD for n=3. Identities of genes are: *Lox* (lipoxygenase TomloxC); *Hdr* (hydroxymethylbutenyl diphoshate reductase); *Dxs* (deoxyxylulose 5-phosphate synthase); *Psy* (phytoene synthase); *Ccd* (carotenoid cleavage dioxygenase). **Figure S6.** Statistical analyses of q-PCR data for nine differentially regulated genes in three ripening stages green (G), breaker (B) and turning (T). The y axis represents ∆CT value (Ct gene-Ct ubiquitin). Data are mean ± SD for n = 3. One-way ANOVA (Tukey’s posthoc test) was used for data analysis and asterisks indicate statistical significance at p<0.05. Identities are as follows: Solyc01g008540.2.1 (Cinnamoyl CoA reductase-like protein); Solyc02g078400.2 (Allantoinase); Solyc02g076920.2.1 (Transcription factor MYC2); Solyc10g005080.2.1 (Late elongated hypocotyl and circadian clock associated-1-like); Solyc09g066410.1.1 (Inorganic phosphate transporter); Solyc06g062540.2.1 (Putative acid phosphatase); Solyc03g083910.2.1 (Acid beta-fructofuranosidase); Solyc01g080280.2.1 (Glutamine synthetase); Solyc10g055390.1 (Nodulin family protein). (PDF 1 MB)

Additional file 3: Table S3: Genes identified as differentially regulated in MYC fruit in comparison with CONT fruit. Genes are ranked in descending order of their log_2_ fold change value. (XLSX 81 KB)
