# Supplementary material for: From root to fruit: RNA-Seq analysis shows that arbuscular mycorrhizal symbiosis may affect tomato fruit metabolism
Source: BMC Genomics. 2014 Mar 21;15(1):221. doi: 10.1186/1471-2164-15-221 (PMC3997964; doi:10.1186/1471-2164-15-221)
Supplement: Supplementary file 1 — Additional file 1: Table S1: Mineral content (C, N, K, P, S) of fully ripe CONTROL and MYC tomato fruit. Values are means ± SD (n = 5). DW, dry weight. Data highlighted in bold are significantly different (Non parametric test Kruskal-Wallis p < 0.05). Table S2. Phenology and fruit yield of CONT and MYC plants: length of the vegetative period (expressed in days after transplanting, DAT), number of days from flowering to the first fruit, fruiting period length, average fruit number per plant, total fruit production, average fruit weight and total fruit yield. Data are expressed as mean ± standard deviation, n = 10. Statistically significant data are highlighted in bold according to the non-parametric Kruskal-Wallis test (p < 0.05). Table S4. Biochemical and nutraceutical parameters measured in fully ripe CONTROL and MYC tomato fruits. Data highlighted in bold are significantly different (one-way ANOVA with Tukey’s posthoc test, p < 0.05). Table S5. Genes and corresponding primers used in real-time RT-PCR. (PDF 83 KB) [file 12864_2013_7042_MOESM1_ESM.pdf]

	CONTROL	*MYC*
C (%)	40.29 ± 0.81	**39.42 ± 0.56**
N (%)	2.23 ± 0.17	**1.29 ± 0.54**
K (mg/g DW)	45.86 ± 1.78	47.97 ± 4.57
P (mg/g DW)	3.20 ± 1.10	2.63 ± 0.57
S (mg/g DW)	1.70 ± 0.68	1.66 ± 0.34

Supplemental Table 1. Mineral content (C, N, K, P, S) of fully ripe CONTROL and MYC tomato fruit. Values are means ± SD (n=5). DW, dry weight. Data highlighted in bold are significantly different (Non parametric Kruskal-Wallis test, $p < 0.05$).

Phenology and fruit yield of high fertilized control plants and low fertilized mycorrhizal plants

	CONTROL	*MYC*
Vegetative period (DAT)	53.27 ± 3.82	52.27 ± 3.45
Flowering-First fruit (days)	15.50 ± 5.88	17.33 ± 4.29
Fruiting period (days)	53.67 ± 14.47	**104.31 ± 21.48**
Average fruit number/plant	2.00 ± 1.41	5.85 ± 2.34
Total fruit production (n°)	20	88
Average fruit weight (g)	23.35 ± 1.74	26.31 ± 1.16
Total fruit yield (g)	467	2315.28

Supplemental Table 2. Phenology and fruit yield of CONT and MYC plants: length of the vegetative period (expressed in days after transplanting, DAT), number of days from flowering to the first fruit, fruiting period length, average fruit number per plant, total fruit production, average fruit weight and total fruit yield. Data are expressed as mean ± standard deviation, n=10. Statistically significant data are highlighted in bold according to the non-parametric Kruskal-Wallis test ($p < 0.05$).

	CONTROL	*MYC*
Radical scavenging activity (µmol trolox/100g fm)	100.89 ± 3.67	127.49 ± 47.29
Reducing activity (µmol trolox/100g fm)	106.68 ± 15.43	**161.11 ± 33.84**
Total phenols (mg GAE/100g fm)	30.35 ± 5.75	41.50 ± 14.72
β-Carotene (mg/100g fm)	4.25 ± 2.85	1.88 ± 0.41
Lycopene (mg/100g fm)	6.51 ± 2.69	4.55 ± 0.95

Supplemental Table 4. Biochemical and nutraceutical parameters measured in fully ripe CONTROL and MYC tomato fruit. Data highlighted in bold are significantly different (one-way ANOVA with Tukey's posthoc test, $p < 0.05$).

Supplemental table 5. Genes and corresponding primers used in real-time RT-PCR

Gene	Primer sequence
Ubiquitin	UbiF:ACCAAGCCAAAGAAGATCAAGC<br>UbiR:GTGAGCCCACACTTACCACAGT
Cinnamoyl CoA reductase-like protein	PaaF:TGGTCAGCCTCGGACACCTGA<br>PaaR:CACGCAGCATCCTCAGCCAATG
Allantoinase	AlnF:GGCAACCTTTGTGGGCGGAAA<br>AlnR:TGCCATGTGGTCGTCTCCAACC
Late elongated hypocotyl and circadian clock associated-1-like	LehF:GGCGGGAAGCAGGTAGATCG<br>LehR:CCGATTCGCAGCCTCTCCAG
Myc2 transcription factor	BhlhF:CAGGGCGTCCGTTTGTTGTG<br>BhlhR:TGGCACATCCTCCTCCTCCA
Inorganic phosphate transporter	IptF:TGGACCCAACGCCACCACAT<br>IptR:TGCACCAACCATTGCCCCTACC
Putative acid phosphatase	PtaseF:GCGATTTCTGCCCCAGTTTG<br>PtaseR:CACTCGTGGACCCCTGCTTT
Glutamine synthetase	GsF:CGCCGCCCAGCTTCAAACAT<br>GsR:CCTCAAGGGTTGGCTCCCACA
Acid beta-fructofuranosidase	AivF:AGGCATTTTGGGACCATTTG<br>AivR:GTCTCTGCACGACCATCAGC
Nodulin family protein	NodulinF:CCTTCCCCTCGGCTCATTTC<br>NodulinR:GTGTTGCCACCACTCGCTGT
Deoxyxylulose 5-phosphate synthase	DXSrtf:GCTTCCGGCTGGAAACAAAGG<br>DXSrtr:CCGCGGGATTCTAGCACAATAG
Hydroxymethylbutenyl diphosphate reductase	HDRrtf:CGACAAACGCAATTCGCTCAC<br>HDRrtr:CGGAGCATCTGATGACGGAG
Phytoene synthase	PSYrtf:TGAGGCAGGCAGCCTTGGTG<br>PSYrtr:GCCCAAATTCCCCGGAATAGG
Lipoxygenase TomloxC	Loxrtf:TTGCCTATGGTGCTGAATGG<br>Loxrtr: TGGATCTTCCTCTGCCAATC
Carotenoid cleavage dioxygenase	CCDrtf:TGACCCCACAAAGAAGGCTCG<br>CCDrtr:CCCAAGCATTGGCGTTGTGG
