# Supplementary material for: From root to fruit: RNA-Seq analysis shows that arbuscular mycorrhizal symbiosis may affect tomato fruit metabolism
Source: BMC Genomics. 2014 Mar 21;15(1):221. doi: 10.1186/1471-2164-15-221 (PMC3997964; doi:10.1186/1471-2164-15-221)
Supplement: Supplementary file 2 — Additional file 2: Figure S1: Mycorrhization parameters of tomato roots inoculated with the AM fungus Funneliformis mosseae. F%: colonization frequency; M%: mycorrhizal colonization intensity; A%: arbuscule abundance; a%: arbuscule abundance in the mycorrhized portion. Mycorrhization parameters were determined according to Trouvelot method (1986). Values are means ± SD (n=5). For each plant, 100 cm of root were measured. Figure S2. Classification of tomato fruit expressed genes. All expressed genes (cutoff RPKM>0.1) are represented. The number of transcripts assigned to tomato biological process (GOSLIM plant mapping) is shown in parentheses. Figure S3. Transcriptional changes induced by mycorrhization in tomato fruits. Normalized expression mean values are plotted versus log2 fold changes and called DEGs (FDR<0.1) are plotted in red. Blue horizontal lines indicate a two-fold change in expression values for up- and down-regulated genes. Figure S4. Sesquiterpenoid phytoalexin biosynthesis pathway. Figure S5. q-PCR data for five selected genes involved in carotenoid metabolism on fruits from mycorrhizal and control plants. The y axis represents ∆CT value (Ct gene-Ct ubiquitin). Data are mean ± SD for n=3. Identities of genes are: Lox (lipoxygenase TomloxC); Hdr (hydroxymethylbutenyl diphoshate reductase); Dxs (deoxyxylulose 5-phosphate synthase); Psy (phytoene synthase); Ccd (carotenoid cleavage dioxygenase). Figure S6. Statistical analyses of q-PCR data for nine differentially regulated genes in three ripening stages green (G), breaker (B) and turning (T). The y axis represents ∆CT value (Ct gene-Ct ubiquitin). Data are mean ± SD for n = 3. One-way ANOVA (Tukey’s posthoc test) was used for data analysis and asterisks indicate statistical significance at p<0.05. Identities are as follows: Solyc01g008540.2.1 (Cinnamoyl CoA reductase-like protein); Solyc02g078400.2 (Allantoinase); Solyc02g076920.2.1 (Transcription factor MYC2); Solyc10g005080.2.1 (Late elongated hypocot [file 12864_2013_7042_MOESM2_ESM.pdf]

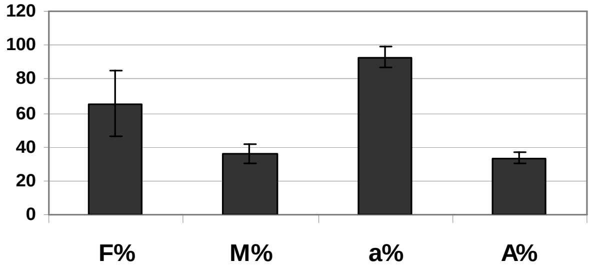

Supplemental Figure 1. Mycorrhization parameters of tomato roots inoculated with the AM fungus *Funneliformis mosseae*. F%: frequency of colonization in the root system; M%: intensity of the mycorrhizal colonization in the root system; A%: arbuscule abundance in the root system; and a%: arbuscule abundance in the mycorrhizal root part. Mycorrhization parameters were determined at the end of the experiment according to Trouvelot method (1986). Values are means ± SD (n=5). For each plant, 100 cm of root were measured.

Sequence distribution: biological_process(Filtered by #Seqs: cutoff=1.0)

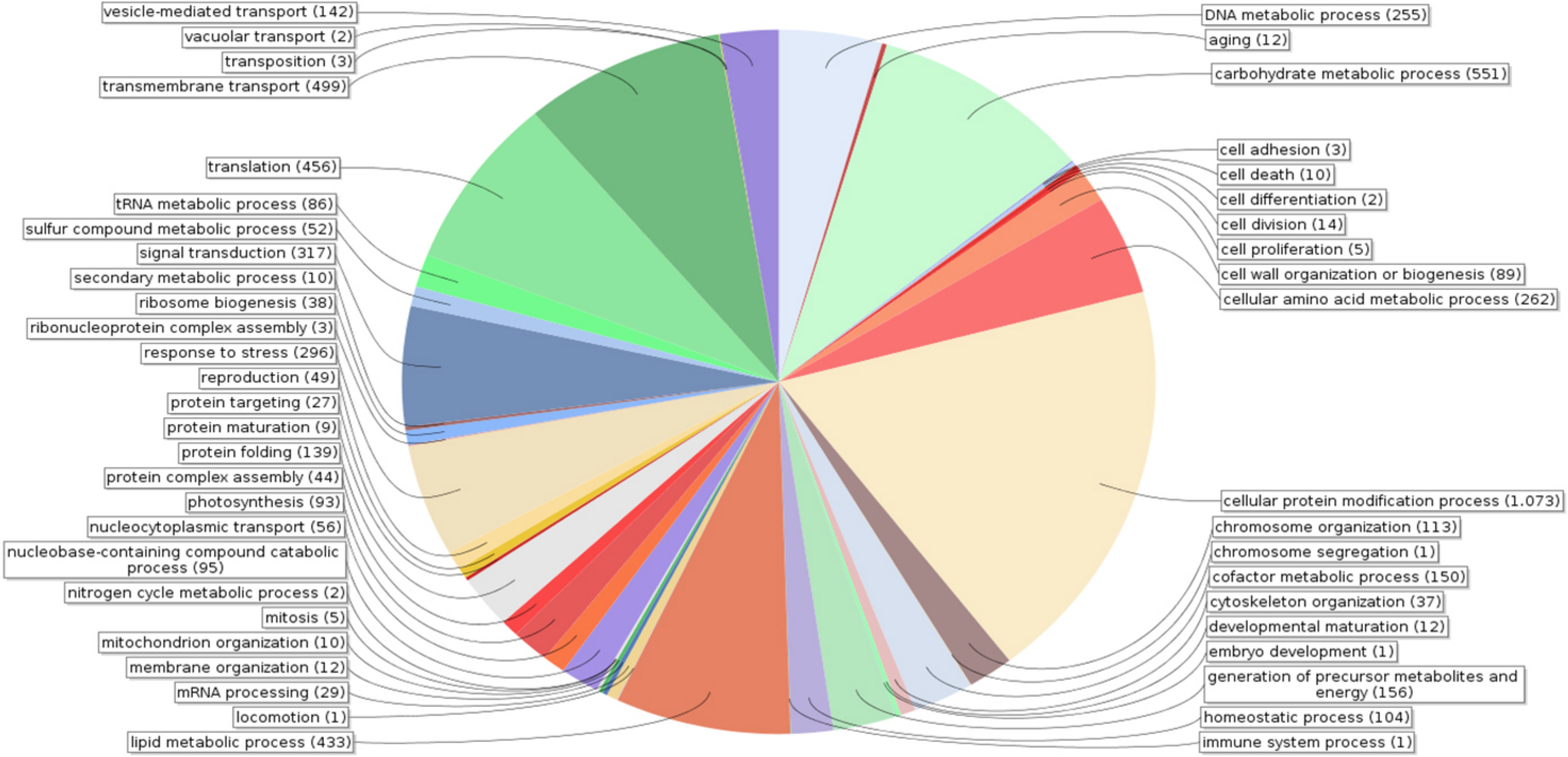

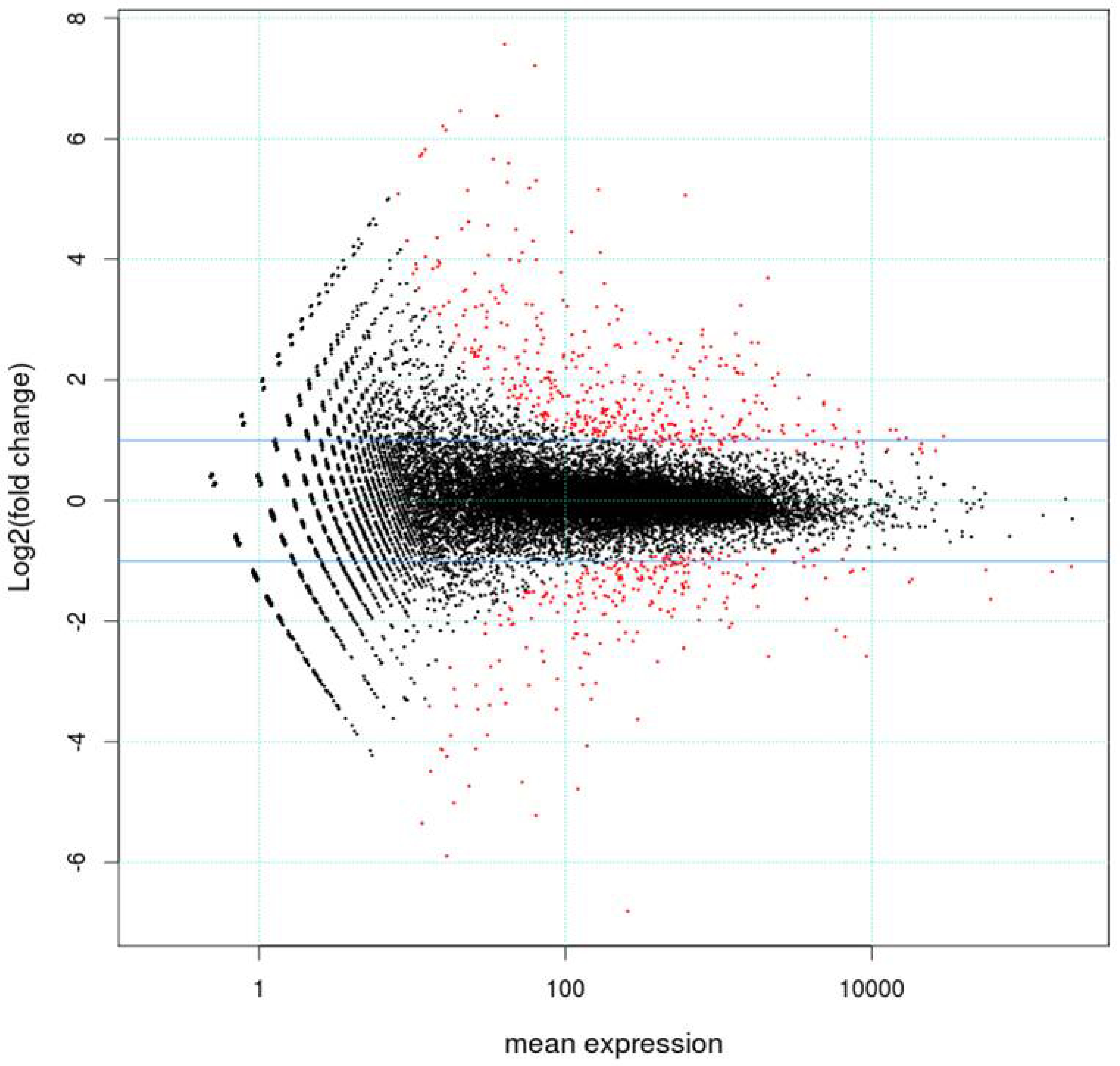
Log2(fold change)
8
6
4
2
0
-2
-4
-6
1
100
10000
mean expression

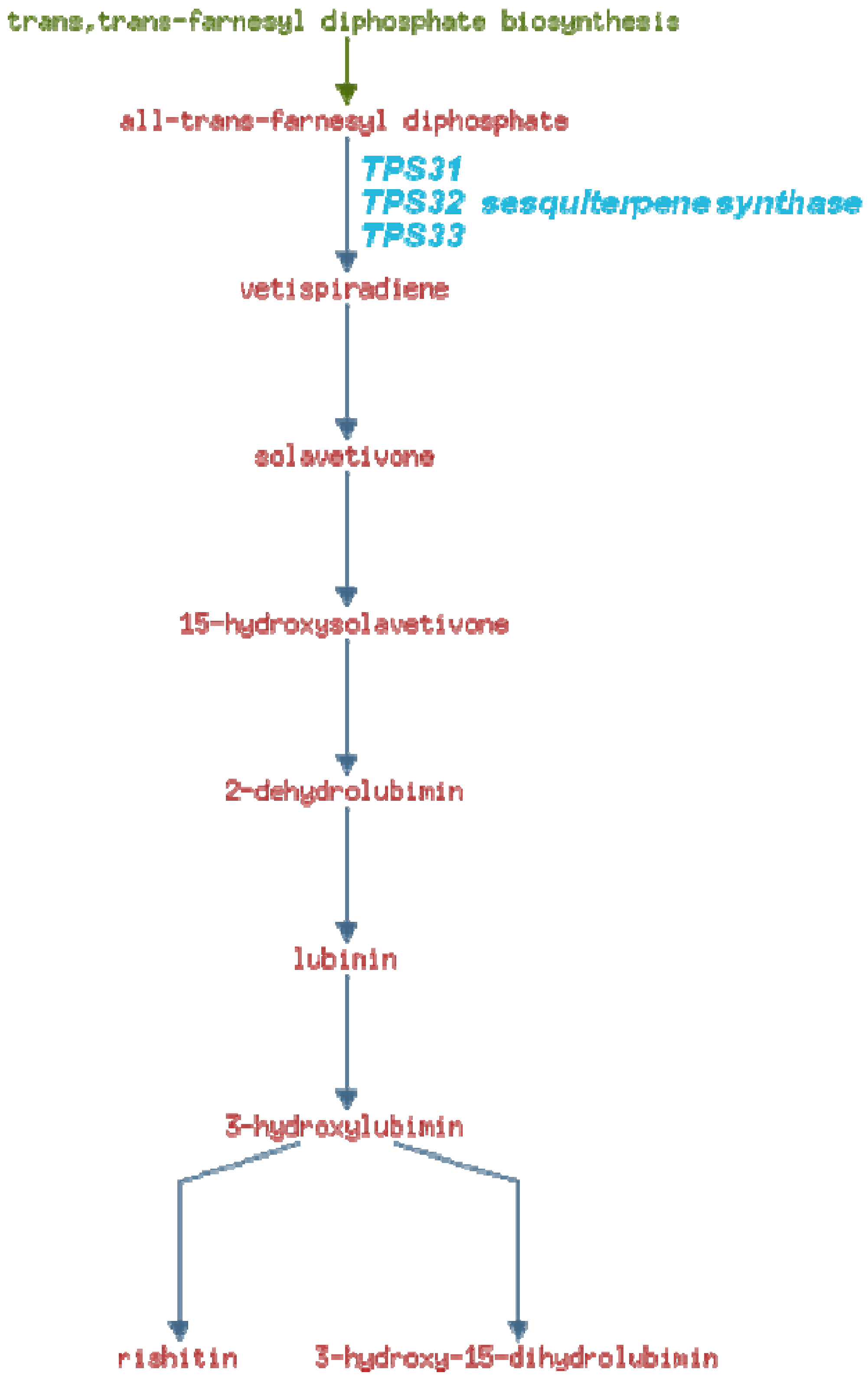

Supplemental figure 4. Sesquiterpenoid phytoalexins biosynthesis pathway.

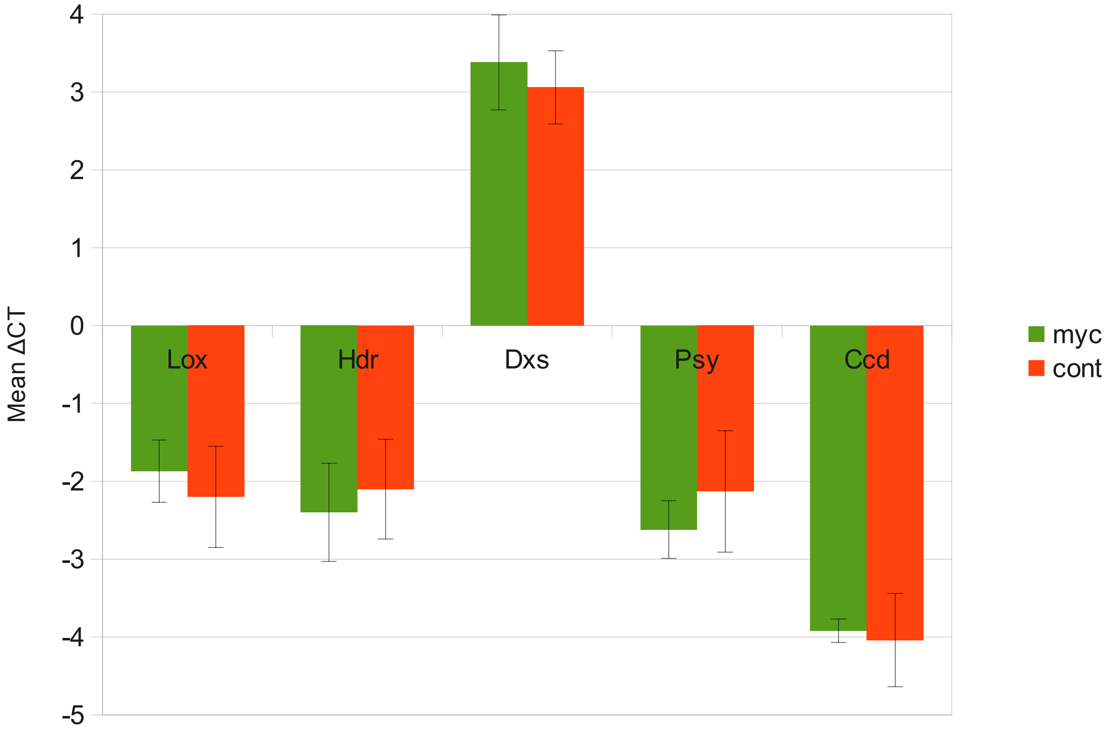

Supplemental Figure 5. q-PCR data obtained for five selected genes involved in the carotenoid metabolism on fruits collected from mycorrhizal and control plants. The y axis represents ΔCT value (Ct gene-Ct ubiquitin). Data are presented as the mean ± SD for n = 3. Identities of genes are as follows: *Lox* (lipoxygenase TomloxC); *Hdr* (hydroxymethylbutenyl diphoshate reductase); *Dxs* (deoxyxylulose 5-phosphate synthase); *Psy* (phytoene synthase); *Ccd* (carotenoid cleavage dioxygenase).

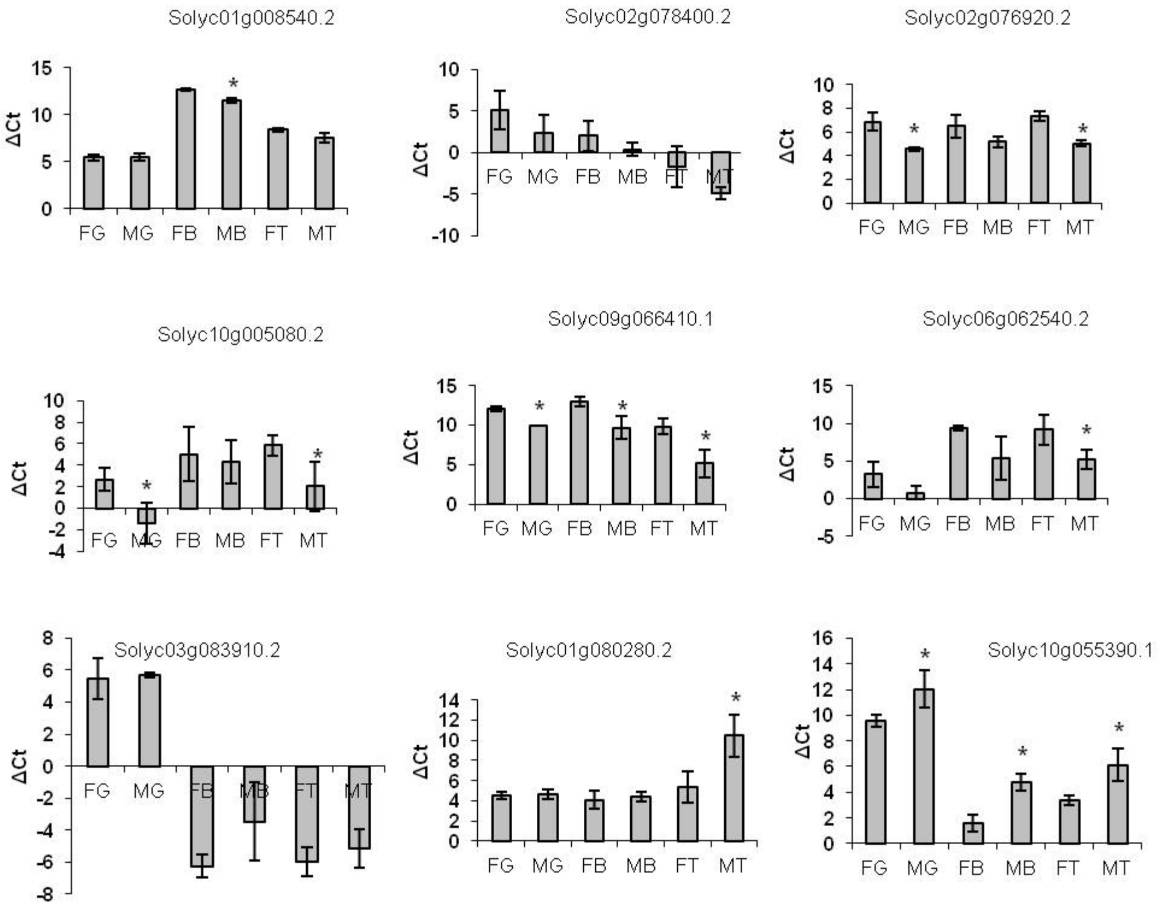

Supplemental figure 6. Statistical analyses of q-PCR data obtained for nine selected differentially regulated genes between mycorrhizal (M) and control (F) plants in three ripening stages green (G), breaker (B) and turning (T). The y axis represents ΔCT value (Ct gene-Ct ubiquitin). Data are presented as the mean ± SD for n = 3. One-way ANOVA (Tukey's posthoc test) was used for data analysis and asterisks indicate statistical significance at $p < 0.05$. Identities of genes are as follows: Solyc01g008540.2.1 (Cinnamoyl CoA reductase-like protein); Solyc02g078400.2 (Allantoinase); Solyc02g076920.2.1 (Transcription factor MYC2); Solyc10g005080.2.1 (Late elongated hypocotyl and circadian clock associated-1-like); Solyc09g066410.1.1 (Inorganic phosphate transporter); Solyc06g062540.2.1 (Putative acid phosphatase); Solyc03g083910.2.1 (Acid beta-fructofuranosidase); Solyc01g080280.2.1 (Glutamine synthetase); Solyc10g055390.1 (Nodulin family protein).
